# Quantum simulation of exact electron dynamics can be more efficient than classical mean-field methods

**DOI:** 10.1038/s41467-023-39024-0

**Published:** 2023-07-10

**Authors:** Ryan Babbush, William J. Huggins, Dominic W. Berry, Shu Fay Ung, Andrew Zhao, David R. Reichman, Hartmut Neven, Andrew D. Baczewski, Joonho Lee

**Affiliations:** 1 Google Quantum AI, Venice, CA USA; 2 Department of Physics and Astronomy, Macquarie University, Sydney, NSW Australia; 3 Department of Chemistry, Columbia University, New York, NY USA; 4 Department of Physics and Astronomy, University of New Mexico, Albuquerque, NM USA; 5 Quantum Algorithms and Applications Collaboratory, Sandia National Laboratories, Albuquerque, NM USA; 6 Present Address: Department of Chemistry and Chemical Biology, Harvard University, Cambridge, USA

**Keywords:** Quantum simulation, Quantum chemistry

## Abstract

Quantum algorithms for simulating electronic ground states are slower than popular classical mean-field algorithms such as Hartree–Fock and density functional theory but offer higher accuracy. Accordingly, quantum computers have been predominantly regarded as competitors to only the most accurate and costly classical methods for treating electron correlation. However, here we tighten bounds showing that certain first-quantized quantum algorithms enable exact time evolution of electronic systems with exponentially less space and polynomially fewer operations in basis set size than conventional real-time time-dependent Hartree–Fock and density functional theory. Although the need to sample observables in the quantum algorithm reduces the speedup, we show that one can estimate all elements of the *k*-particle reduced density matrix with a number of samples scaling only polylogarithmically in basis set size. We also introduce a more efficient quantum algorithm for first-quantized mean-field state preparation that is likely cheaper than the cost of time evolution. We conclude that quantum speedup is most pronounced for finite-temperature simulations and suggest several practically important electron dynamics problems with potential quantum advantage.

## Abstract (web summary)

It is often assumed that systems that can be analyzed accurately via mean-field theory would not be worth looking at using quantum algorithms, given entanglement plays no key role. Here, the authors show instead that a quantum advantage can be expected for simulating the exact time evolution of such electronic systems.

## Introduction

Quantum computers were first proposed as tools for dynamics by Feynman^1^ and later shown to be universal for that purpose by Lloyd et al.^2^. Like those early papers, most work on this topic assumes that the advantage of quantum computers for dynamics is that they provide an approach to simulation with systematically improvable precision but without scaling exponentially. Here, we advance and analyze a different idea: certain (exact) quantum algorithms for dynamics may be more efficient than even classical methods that make uncontrolled approximations. By exact we mean that the time-evolved quantum state differs in 1-norm from the full configuration interaction dynamics within a basis by at most *ϵ*, regardless of the initial state, with a refinement cost scaling as ${{{{{{{\mathcal{O}}}}}}}}(\log (1/\epsilon ))$. We examine this in the context of simulating interacting fermions—systems of relevance in fields such as chemistry, physics, and materials science.

It is often the case that practically relevant ground-state problems in chemistry and materials science do not exhibit a strong correlation. For those problems, many classical heuristic methods work well^3–5^. Even for some strongly correlated systems, there are successful polynomial-scaling classical methods^6^. Here, we argue that even if electronic systems are well described by mean-field theory, quantum algorithms can achieve speedup over classical algorithms for simulating the time evolution of such systems. We focus on comparing to mean-field methods such as real-time time-dependent Hartree–Fock and density functional theory due to their popularity and well-defined scaling. Nonetheless, many of our arguments translate to advantages over other classical approaches to dynamics that are more accurate and expensive than mean-field methods. This is a sharp contrast to prior studies of quantum algorithms, which have focused on strongly correlated ground state problems such as FeMoCo^7–11^, P450^12^, chromium dimers^13^ and jellium^14–17^, assessing quantum advantage over only the most accurate and costly classical algorithms.

Quantum algorithms competitive with classical algorithms for efficient-to-simulate quantum dynamics have been analyzed in contexts outside of fermionic simulation. For example, work by Somma^18^ showed that certain one-dimensional quantum systems, such as harmonic oscillators, could be simulated with sublinear complexity in system size. Experimentally motivated work by Geller et al.^19^ also proposed simulating quantum systems in a single-excitation subspace, a task for which they suggested a constant factor speedup was plausible. However, neither work is connected to the context studied here.

In this work, we begin by analyzing the cost of classical mean-field dynamics and recent exact quantum algorithms in first quantization, focusing on why there is often a quantum speedup in the number of basis functions over classical mean-field methods. Next, we analyze the overheads associated with measuring quantities of interest on a quantum computer and introduce more efficient methods for measuring the one-particle reduced density matrix in the first quantization (which characterizes all mean-field observables). Then, we discuss the costs of preparing mean-field states on the quantum computer and describe new methods that make this cost likely negligible compared to the cost of time evolution. We conclude with a discussion of systems where these techniques might lead to a practical quantum advantage over classical mean-field simulations.

## Results

### Classical mean-field dynamics

Here we will discuss mean-field classical algorithms for simulating the dynamics of interacting systems of electrons and nuclei. Thus, we will focus on the ab initio Hamiltonian with *η* particles discretized using *N* basis functions, which can be expressed as

1 $$H=\mathop{\sum }\limits_{\mu \nu }^{N}{h}_{\mu \nu }{a}_{\mu }^{{{{\dagger}}} }{a}_{\nu }+\frac{1}{2}\mathop{\sum }\limits_{\mu \nu \lambda \sigma }^{N}\left(\mu \nu|\lambda \sigma \right){a}_{\mu }^{{{{\dagger}}} }{a}_{\lambda }^{{{{\dagger}}} }{a}_{\sigma }{a}_{\nu }$$

where ${a}_{\mu }^{({{{\dagger}}} )}$ is the fermionic annihilation (creation) operator for the *μ*-th orbital and integral values are given by

2 $${h}_{\mu \nu }=\int\,{{{{{{{\rm{d}}}}}}}}r\,{\phi }_{\mu }^{*}\left(r\right)\left(-\frac{{\nabla }^{2}}{2}+V\left(r\right)\right){\phi }_{\nu }\left(r\right),$$

3 $$\left(\mu \nu|\lambda \sigma \right)=\int\,{{{{{{{\rm{d}}}}}}}}{r}_{1}{{{{{{{\rm{d}}}}}}}}{r}_{2}\frac{{\phi }_{\mu }^{*}\left({r}_{1}\right){\phi }_{\nu }\left({r}_{1}\right){\phi }_{\lambda }^{*}\left({r}_{2}\right){\phi }_{\sigma }\left({r}_{2}\right)}{\left|{r}_{1}-{r}_{2}\right|}\,.$$

Here, *V*(*r*) is the external potential (perhaps arising from the nuclei) and *ϕ*_*μ*_(*r*) represents a spatial orbital.

Exact quantum dynamics is encoded by the time-dependent Schrödinger equation given by

4 $$i\frac{\partial }{\partial t}\left|\psi \left(t\right)\right\rangle=H\left|\psi \left(t\right)\right\rangle \,.$$

Mean-field dynamics, such as real-time time-dependent Hartree–Fock (RT-TDHF)^20^, employs a time-dependent variational principle within the space of single Slater determinants (i.e., anti-symmetrized product states) to approximate Eq. (4). Other methods with similar cost such as real-time time-dependent density functional theory (RT-TDDFT) rely on a relationship between the interacting system and an auxiliary non-interacting system to define dynamics within a space of single Slater determinants^20–22^. In both methods, there are *η* occupied orbitals, each expressed as a linear combination of *N* basis functions using the coefficient matrix, **C**_occ_. The dimension of **C**_occ_ is *N* × *η*. These orbitals constitute a Slater determinant, requiring ${{{{{{{\mathcal{O}}}}}}}}(N\eta \log (1/\epsilon ))$ space for classical storage.

As a result of this approximation, we solve the following effective time-dependent equation for the occupied orbital coefficients that specify the Slater determinant **C**_occ_(*t*) at a given moment in time:

5 $$i\frac{\partial {{{{{{{{\bf{C}}}}}}}}}_{{{{{{{{\rm{occ}}}}}}}}}\left(t\right)}{\partial t}={{{{{{{\bf{F}}}}}}}}\left(t\right){{{{{{{{\bf{C}}}}}}}}}_{{{{{{{{\rm{occ}}}}}}}}}\left(t\right)$$

where the effective one-body mean-field operator **F**(*t*), also known as the time-dependent Fock matrix, is

6 $${F}_{\mu \nu }\,\left(t\right)={h}_{\mu \nu }+\mathop{\sum }\limits_{\lambda \sigma }^{N}\left(\left(\mu \nu|\lambda \sigma \right)-\frac{\left(\mu \sigma|\lambda \nu \right)}{2}\right){P}_{\sigma \lambda }\,\left(t\right)$$

with ${{{{{{{\bf{P}}}}}}}}(t)={{{{{{{{\bf{C}}}}}}}}}_{{{{{{{{\rm{occ}}}}}}}}}(t){({{{{{{{{\bf{C}}}}}}}}}_{{{{{{{{\rm{occ}}}}}}}}}(t))}^{{{{\dagger}}} }$. While **F**(*t*) is an *N* × *N* dimensional matrix, we can apply it to **C**_occ_(*t*) without explicitly constructing it, thus avoiding a space complexity of ${{{{{{{\mathcal{O}}}}}}}}({N}^{2}\log (1/\epsilon ))$. Using the most common methods of applying this matrix to update each of *η* occupied orbitals in **C**_occ_(*t*) requires $\widetilde{{{{{{{{\mathcal{O}}}}}}}}}({N}^{2}\eta )$ total operations. (Throughout the paper we use the convention that $\widetilde{{{{{{{{\mathcal{O}}}}}}}}}(\cdot )$ implies suppressing polylogarithmic factors).

However, a recent technique referred to as occ-RI-K by Head-Gordon and co-workers^23^, and similarly Adaptively Compressed Exchange (ACE)^24,25^ by Lin and co-workers, further reduces this cost. These methods leverage the observation that, when restricted to the subspace of the *η* occupied orbitals, the effective rank of the Fock operator scales as ${{{{{{{\mathcal{O}}}}}}}}(\eta )$. This gives an approach to updating the Fock operator that requires only

7 $$\widetilde{{{{{{{{\mathcal{O}}}}}}}}}(N\,{\eta }^{2})$$

operations. Below we will use gate complexity and the number of operations interchangeably when discussing the scaling of classical algorithms. Although these techniques are not implemented in every quantum chemistry code, we regard them as the main point of comparison to quantum algorithms. We also note that RT-TDDFT with hybrid functionals^26^ has the same scaling as RT-TDHF. Simpler RT-TDDFT methods (i.e., those without exact exchange) can achieve better scaling, $\widetilde{{{{{{{{\mathcal{O}}}}}}}}}(N\eta )$ in a plane wave basis, but are often less accurate.

For finite-temperature simulation, one often needs to track *M* > *η* orbitals with appreciable occupations, increasing the space complexity to ${{{{{{{\mathcal{O}}}}}}}}(NM\log (1/\epsilon ))$. This increases the cost of occ-RI-K or ACE mean-field updates to $\widetilde{{{{{{{{\mathcal{O}}}}}}}}}(N{M}^{2})$. At temperatures well above the Fermi energy, most orbitals have appreciable occupations so *M* ≃ *N*. More expensive methods for dynamics that include electron correlation in the dynamics tend to scale at least linearly in the cost of ground state simulation at that level of theory. Thus, speedup over mean-field methods implies speedup over more expensive methods.

In recent years, by leveraging the nearsightedness of electronic systems^27^, linear-scaling methods have been developed that achieve updates scaling as ${{{{{{{\mathcal{O}}}}}}}}(N)$^28^. For RT-TDHF and RT-TDDFT, linear scaling comes from the fact that the off-diagonal elements of **P** fall off quickly with distance for the ground state^29^ and some low-lying excited states^30^ in a localized basis. One can show that for gapped ground states, the decay rate is exponential, whereas for metallic ground states, it is algebraic^27^. However, often such asymptotic behavior only onsets for very large systems, and the onset can be highly system-dependent. This should be contrasted with the scaling analyzed above and the scaling of quantum algorithms (*vide infra*) that onsets already at modest system sizes. Furthermore, the nearsightedness of electrons does not necessarily hold for dynamics of highly excited states and at high temperatures. It has also been suggested that one can exploit a low-rank structure of occupied orbitals using the quantized tensor train format^31^. Assuming the compression of orbitals in real space is efficient such that the rank does not grow with system size or the number of grid points, the storage cost is reduced to $\tilde{{{{{{{{\mathcal{O}}}}}}}}}(\eta )$, and the update cost is $\tilde{{{{{{{{\mathcal{O}}}}}}}}}({\eta }^{2})$. It is unclear how well compression can be realized for dynamics problems and finite-temperature problems, and to our knowledge, it has never been deployed for those purposes. Due to these limitations, we do not focus on comparing quantum algorithms to classical linear-scaling methods or quantized tensor train approaches.

We now discuss how many time steps are required to perform time evolution using classical mean-field approaches. The number of time steps will depend on the target precision as well as the total unitless time

8 $$T=\mathop{\max }\limits_{{{{{{{{\bf{{C}}}}}}}_{{{{{{{{\rm{occ}}}}}}}}}}}}\left|{{{{{{{\bf{F}}}}}}}}\right|\,t,$$

where *t* is the duration of time-evolution and ∥⋅∥ denotes the spectral norm. This dependence on the norm of **F** is similar to what would be obtained in the case of linear differential equations despite the dependence on **C**_occ_; see Supplementary Note 1 for a derivation. We can upper bound *T* by considering its scaling on a local basis, and with open boundary conditions. We find

9 $$\mathop{\max }\limits_{{{{{{{{\bf{{C}}}}}}}_{{{{{{{{\rm{occ}}}}}}}}}}}}\,\left|{{{{{{{\bf{F}}}}}}}}\right|\,={{{{{{{\mathcal{O}}}}}}}}\,\left(\frac{{\eta }^{2/3}}{\delta }\,+\frac{1}{{\delta }^{2}}\right)\,={{{{{{{\mathcal{O}}}}}}}}\,\left({N}^{1/3}{\eta }^{1/3}\,+\frac{{N}^{2/3}}{{\eta }^{2/3}}\right),$$

where $\delta={{{{{{{\mathcal{O}}}}}}}}({(\eta /N)}^{1/3})$ is the minimum grid spacing when taking cell volume proportional to *η*. The first term comes from the Coulomb operator, and the second comes from the kinetic energy operator.

We briefly describe how this scaling for the norm is obtained and refer the reader to Supplementary Note 1 for more details. The 1/*δ*^2^ term is obtained from the kinetic energy term in *h*_*μν*_. When diagonalized, that term will be non-zero only when *μ* = *ν* with entries scaling as ${{{{{{{\mathcal{O}}}}}}}}(1/{\delta }^{2})$ due to the ∇^2^ in the expression for *h*_*μν*_. That upper bounds the spectral norm for this diagonal matrix, and the spectral norm is unchanged under a change of basis. The *η*^2/3^/*δ* comes from the sum in the expression for *F*_*μν*_. To bound the tensor norm of (*μν*∣*λσ*) − (*μσ*∣*λν*)/2 we can bound the norms of the two terms separately. For each, the tensor norm can be upper bounded by noting that the summing over *μν*, *λσ* with normalized vectors corresponds to transformations of the individual orbitals in the integral defining (*μν*∣*λσ*). Since orbitals cannot be any more compact than width *δ*, the 1/∣*r*_1_ − *r*_2_∣ in the integral averages to give ${{{{{{{\mathcal{O}}}}}}}}(1/\delta )$. There is a further factor of *η*^2/3^ when accounting for *η* electrons that cannot be any closer than *η*^1/3^*δ* on average.

The number of time steps required to effect evolution to within error *ϵ* depends on the choice of time integrator. Many options are available^32–34^, and the optimal choice depends on implementation details like the basis set and pseudization scheme, as well as the desired accuracy^35^. In Supplementary Note 1, we argue that the minimum number of time steps *t*/Δ*t* one could hope for by using an arbitrarily high order integration scheme of this sort is *T*^1+*o*(1)^/*ϵ*^*o*(1)^. In particular, for an order *k* integrator, the error can be bounded as ${{{{{{{\mathcal{O}}}}}}}}({(\parallel {{{{{{{\bf{F}}}}}}}}\parallel {{\Delta }}t)}^{k+1})$, with a possibly *k*-dependent constant factor that is ignored in this expression. That means the error for *t*/Δ*t* time steps is ${{{{{{{\mathcal{O}}}}}}}}(t\parallel {{{{{{{\bf{F}}}}}}}}{\parallel }^{k+1}{{\Delta }}{t}^{k})$. To obtain error no more than *ϵ*, take $({t/{{\Delta }}t})^{k}={{{{{{{\mathcal{O}}}}}}}}\left(\right.(t\parallel {{{{{{{\bf{F}}}}}}}}{\parallel }^{k+1}/\epsilon )$, so the number of time steps is $t/{{\Delta }}t={{{{{{{\mathcal{O}}}}}}}}({T}^{1+1/k}/{\epsilon }^{1/k})$. Plugging Eq. (9) into Eq. (8) and multiplying the update cost in Eq. (7) by *T*^1+*o*(1)^/*ϵ*^*o*(1)^ time steps, we find the number of operations required for classical mean-field time-evolution is

10 $$\left({N}^{4/3}{\eta }^{7/3}t+{N}^{5/3}{\eta }^{4/3}t\right){\left(\frac{Nt}{\epsilon }\right)}^{o\left(1\right)}\,.$$

Finally, when performing mean-field dynamics, the central quantity of interest is often the one-particle reduced density matrix (1-RDM). The 1-RDM is an *N* × *N* matrix defined as a function of time with matrix elements

11 $${\rho }_{\mu \nu }\left(t\right)=\left\langle \psi \left(t\right)\right|{a}_{\mu }^{{{{\dagger}}} }{a}_{\nu }\left|\psi \left(t\right)\right\rangle .$$

The 1-RDM is the central quantity of interest because it can be used to reconstruct any observable associated with a Slater determinant efficiently. For more general states, one would also need higher order RDMs; however, all higher order RDMs can be exactly computed from the 1-RDM via Wick’s theorem when the wavefunction is a single Slater determinant^36^. Thus, when mean-field approximations work well, the time-dependent 1-RDM can also be used to compute multi-time correlators such as Green’s functions and spectral functions.

### Exact quantum dynamics in first quantization

One of the key advantages of some quantum algorithms over mean-field classical methods is the ability to perform dynamics using the compressed representation of first quantization. First-quantized quantum simulations date back to refs. ^37–40^. They were first applied to fermionic systems in ref. ^38^ and developed for molecular systems in refs. ^41,42^. In first quantization, one encodes the wavefunction using *η* different registers (one for each occupied orbital), each of size $\log N$ (to index the basis functions comprising each occupied orbital). The space complexity of first-quantized quantum algorithms is ${{{{{{{\mathcal{O}}}}}}}}(\eta \log N)$.

As described previously, mean-field classical methods require space complexity of ${{{{{{{\mathcal{O}}}}}}}}(N\eta \log (1/\epsilon ))$ where *ϵ* is the target precision. Thus, these quantum algorithms require exponentially less space in *N*. Usually, when one thinks of quantum computers more efficiently encoding representations of quantum systems, the advantage comes from the fact that the wavefunction might be specified by a Hilbert space vector of dimension $$\left(\begin{array}{l}N\\ \eta \end{array}\right)$$ and could require as much space to represent explicitly on a classical computer. However, this alone *cannot* give exponential quantum advantage in storage in *N* over classical mean-field methods since mean-field methods only resolve entanglement arising from antisymmetry and do not attempt to represent wavefunction in the full Hilbert space. Instead, the scaling advantage these quantum algorithms have over mean-field methods is related to the ability to store the distribution of each occupied orbital over *N* basis functions, using only $\log N$ qubits. But quantum algorithms require more than the compressed representations of first quantization in order to realize a scaling advantage over classical mean-field methods; they must also have sufficiently low gate complexity.

Here we review and tighten bounds for the most efficient known quantum algorithms for simulating the dynamics of interacting electrons. Early first-quantized algorithms for simulating chemistry dynamics such as refs. ^41,42^ were based on Trotterization of the time-evolution operator in a real space basis and utilized the quantum Fourier transform to switch between a representation where the potential operator was diagonal and the kinetic operator was diagonal. This enabled Trotter steps with gate complexity $\widetilde{{{{{{{{\mathcal{O}}}}}}}}}({\eta }^{2})$ but the number of Trotter steps required for the approach of those papers scaled worse than linearly in *N*, *η*, the simulation time *t* and the desired inverse error in the evolution, 1/*ϵ*.

Leveraging recent techniques for bounding Trotter error^43–45^, in Supplementary Note 2 we show that using sufficiently high-order Trotter formulas, the overall gate complexity of these algorithms can be reduced to

12 $$\left({N}^{1/3}{\eta }^{7/3}t+{N}^{2/3}{\eta }^{4/3}t\right){\left(\frac{Nt}{\epsilon }\right)}^{o\left(1\right)}\,.$$

This is the lowest reported scaling of any Trotter-based first-quantized quantum chemistry simulation. We remark that the *N*^1/3^*η*^7/3^*t* scaling is dominant whenever *N* < Θ(*η*^3^). In that regime, it represents a quartic speedup in basis size for propagation over the classical mean-field scaling given in Eq. (10). While efficient explicit circuits such as those in ref. ^46^ can be used to perform Trotter steps in this representation, more work would be required to determine the constant factors associated with the number of Trotter steps required. Prior analyses of the requisite Trotter number for chemistry have generally found that constant factors are low, but focused on different representations or lower order formulas^16,47–50^.

The first algorithms to achieve sublinear scaling in *N* were those introduced by Babbush et al.^51^. That work focused on first-quantized simulation in a plane wave basis and leveraged the interaction picture simulation scheme of ref. ^52^ to give gate complexity scaling as

13 $$\widetilde{{{{{{{{\mathcal{O}}}}}}}}}\left({N}^{1/3}{\eta }^{8/3}t\right)\,.$$

When *N* > Θ(*η*^4^), this algorithm is more efficient than the Trotter-based approach. Since that is also the regime where the second term in Eq. (10) dominates that scaling, this represents a quintic speedup in *N* and a quadratic slowdown in *η* over mean-field classical algorithms. The work of Su et al.^53^ analyzed the constant factors in the scaling of this algorithm for use in ground state preparation via quantum phase estimation^54^. In Supplementary Note 3 of this work, we analyze the constant factors in the scaling of this algorithm when deployed for time evolution. Su et al.^53^ also introduced algorithms with the same scaling as Eq. (13) but in a grid representation (see Appendix K therein).

A key component of the algorithms of refs. ^51,53^ is the realization of block encodings^55^ with just $\widetilde{{{{{{{{\mathcal{O}}}}}}}}}(\eta )$ gates. The difficult part of block encoding is preparing a superposition state with amplitudes proportional to the square root of the Hamiltonian term coefficients. A novel quantum algorithm is devised in ref. ^51^, which scales only polylogarithmically in basis size. The *N*^1/3^ dependence of Eq. (13) enters via the number of times the block encoding must be repeated to perform time evolution, related to the norm of the potential operator. Suppose one can soften the Coulomb potential while retaining target precision for the simulation. In that case, the norm of the potential term can be reduced to polylogarithmic dependence on *N* (see Supplementary Note 4 for details). In that case, an exponential quantum advantage in *N* is possible.

We note that second-quantized algorithms outperform first-quantized quantum algorithms in gate complexity when *N* < Θ(*η*^2^). This is because while the best scaling Trotter steps in the first quantization require $\widetilde{{{{{{{{\mathcal{O}}}}}}}}}({\eta }^{2})$ gates^42^, the best scaling Trotter steps in the second quantization require $\widetilde{{{{{{{{\mathcal{O}}}}}}}}}(N)$ gates. As recently shown in ref. ^45^, such approaches lead to a total gate complexity for Trotter-based second-quantized algorithms scaling as

14 $$\left({N}^{4/3}{\eta }^{1/3}t+\frac{{N}^{5/3}}{{\eta }^{2/3}}t\right){\left(\frac{Nt}{\epsilon }\right)}^{o\left(1\right)}\,.$$

In the limit that *η* = Θ(*N*), this approach has ${{{{{{{\mathcal{O}}}}}}}}({N}^{5/3})$ gate complexity, which is significantly less than the ${{{{{{{\mathcal{O}}}}}}}}({N}^{8/3})$ gate complexity of Trotter-based first-quantized quantum algorithms mentioned here, or the ${{{{{{{\mathcal{O}}}}}}}}({N}^{11/3})$ gate complexity of classical mean-field algorithms. (See Supplementary Note 5 for a discussion on the overall quantum speedup in different regimes of how *N* scales in *η*.) However, these second-quantized approaches generally require at least ${{{{{{{\mathcal{O}}}}}}}}(N)$ qubits. The approach used in ref. ^45^ to implement Trotter steps involves the fast multipole method^56^, which requires ${{{{{{{\mathcal{O}}}}}}}}(N\log N)$ qubits as well as the restriction to a grid-like basis. When using such basis sets, we expect *N* ≫ *η*, and so this space complexity would be prohibitive for quantum computers.

Methods such as fast multipole^56^, Barnes-Hut^57^, or particle-mesh Ewald^58^ compute the Coulomb potential in time $\widetilde{{{{{{{{\mathcal{O}}}}}}}}}(\eta )$ when implemented within the classical random access memory model. If the Coulomb potential could be computed with that complexity on a quantum computer it would speed up the first-quantized Trotter algorithms discussed here by a factor of ${{{{{{{\mathcal{O}}}}}}}}(\eta )$. However, it is unclear whether such algorithms extend to the quantum circuit model with the same complexity without unfavorable assumptions such as QRAM^59,60^, or without restricting the maximum number of electrons within a region of space (see Supplementary Note 5 for details). Thus, we exclude such approaches from our comparisons here.

### Quantum measurement costs

In contrast to classical mean-field simulations, on a quantum computer, all observables must be sampled from the quantum simulation. There are a variety of techniques for doing this, with the optimal choice depending on the target precision in the estimated observable as well as the number and type of observables one wishes to measure. For example, when measuring *W* unit norm observables to precision *ϵ* one could use algorithms introduced in ref. ^61^ which require $\widetilde{{{{{{{{\mathcal{O}}}}}}}}}(\sqrt{W}/\epsilon )$ state preparations and ${{{{{{{\mathcal{O}}}}}}}}(W\log (1/\epsilon ))$ ancillae. Thus, to measure all $W={{{{{{{\mathcal{O}}}}}}}}({N}^{2})$ elements of the 1-RDM to a fixed additive error in each element, this approach would require $\widetilde{{{{{{{{\mathcal{O}}}}}}}}}(N/\epsilon )$ circuit repetitions. While scaling optimally in *ϵ* for quantum algorithms, this linear scaling in *N* would decrease the speedup over classical mean-field algorithms.

Here we introduce a new classical shadows protocol for measuring the 1-RDM. Classical shadows were introduced in ref. ^62^ and adapted for second-quantized fermionic systems in refs. ^63–66^. Our approach is to apply a separate random Clifford channel to each of the *η* different $\log N$ sized registers representing an occupied orbital. Applying a random Clifford on $\log N$ qubits requires ${{{{{{{\mathcal{O}}}}}}}}({\log }^{2}\,N)$ gates; thus, ${{{{{{{\mathcal{O}}}}}}}}(\eta {\log }^{2}\,N)$ gates comprise the full channel (a negligible cost relative to time-evolution). In Supplementary Note 6, we prove that repeating this procedure $\widetilde{{{{{{{{\mathcal{O}}}}}}}}}(\eta /{\epsilon }^{2})$ times enables the estimation of all 1-RDM elements to within additive error *ϵ*. We also prove that this same procedure allows for estimating all higher-order *k*-particle RDMs elements with $\widetilde{{{{{{{{\mathcal{O}}}}}}}}}({k}^{k}{\eta }^{k}/{\epsilon }^{2})$ circuit repetitions. In the next section and in Supplementary Note 7, we describe a way to map second-quantized representations to first quantization, effectively extending the applicability of these classical shadows techniques to second quantization as well.

To give some intuition for how this works, we consider the 1-RDM elements in first quantization:

15 $${\rho }_{\mu \nu }\left(t\right)=\left\langle \psi \left(t\right)\right|\left(\mathop{\sum }\limits_{j=1}^{\eta }\left|\mu \right\rangle \,\,{\left\langle \nu \right|}_{j}\right)\left|\psi \left(t\right)\right\rangle \,,$$

where the subscript *j* indicates which of the *η* registers the orbital-*ν* to orbital-*μ* transition operator acts upon. Due to the antisymmetry of the occupied orbital registers in first quantization, we could also obtain the 1-RDM by measuring the expectation value of an operator such as $\eta \left|p\right\rangle \,\,{\left\langle q\right|}_{1}$, which acts on just one of the *η* registers. Because $\eta \left|p\right\rangle \,\,{\left\langle q\right|}_{1}$ has the Hilbert–Schmidt norm of ${{{{{{{\mathcal{O}}}}}}}}(\eta )$, the standard classical shadows procedure applied to this $\log N$ sized register would require $\widetilde{{{{{{{{\mathcal{O}}}}}}}}}({\eta }^{2}/{\epsilon }^{2})$ repetitions. But we can parallelize the procedure by also collecting classical shadows on the other *η* − 1 registers simultaneously. One way of interpreting the results we prove in Supplementary Note 6 is that, due to antisymmetry, these registers are anticorrelated. As a result, collecting shadows on all *η* registers simultaneously reduces the overall cost by at least a factor of *η*. To obtain *W* elements of the 1-RDM, one will need to perform an offline classical inversion of the Clifford channel that will scale as $\widetilde{{{{{{{{\mathcal{O}}}}}}}}}(W{\eta }^{2}/{\epsilon }^{2})$; of course, any quantum or classical algorithm for estimating *W* quantities must have gate complexity of at least *W*. However, this only needs to be done once and does not scale in *t*. As a comparison, the cost of computing 1-RDM classically without exploiting sparsity is ${{{{{{{\mathcal{O}}}}}}}}(W\eta )$.

When simulating systems that are well described by mean-field theory, all observables can be efficiently obtained from the time-dependent 1-RDM. However, for observables such as the energy that have a norm growing in system size or basis size, targeting fixed additive error in the 1-RDM elements will not be sufficient for fixed additive error in the observable. In such situations, it could be preferable to estimate the observable of interest directly using a combination of block encodings^55^ and amplitude amplification^67^ (see e.g., ref. ^68^). Assuming the cost of block encoding the observable is negligible to the cost of time-evolution (true for many observables, including energy), this results in needing ${{{{{{{\mathcal{O}}}}}}}}(\lambda /\epsilon )$ circuit repetitions, where *λ* is the 1-norm associated with the block encoding of the observable. For example, whereas there are many correlation functions with $\lambda={{{{{{{\mathcal{O}}}}}}}}(1)$, for the energy $\lambda={{{{{{{\mathcal{O}}}}}}}}({N}^{1/3}{\eta }^{5/3}+{N}^{2/3}{\eta }^{1/3})$^51^. Multiplying that to the cost of quantum time-evolution further reduces the quantum speedup.

The final measurement cost to consider is that of resolving observables in time. In some cases, e.g., when computing scattering cross sections or reaction rates, one might be satisfied measuring the state of the simulation at a single point in time *t*. However, in other situations, one might wish to simulate time-evolution up to a maximum duration of *t*, but sample quantities at *L* different points in time. Most quantum simulation methods that accomplish this goal scale as ${{{{{{{\mathcal{O}}}}}}}}(L)$ (${{{{{{{\mathcal{O}}}}}}}}(Lt)$ in the case where the points are evenly spaced in time). However, the work of ref. ^61^ shows that this cost can be reduced to ${{{{{{{\mathcal{O}}}}}}}}(\sqrt{L}t)$, but with an additional additive space complexity of $\widetilde{{{{{{{{\mathcal{O}}}}}}}}}(L)$. Either way, this is another cost that plagues quantum but not classical algorithms.

### Quantum state preparation costs

Initial state preparation can be as simple or as complex as the state that one desires to begin the simulation in. Since the focus of this paper is outperforming mean-field calculations, we will discuss the cost of preparing Slater determinants within first quantization. For example, one may wish to start in the Hartree–Fock state (the lowest energy Slater determinant). Classical approaches to computing the Hartree–Fock state scale as roughly $\widetilde{{{{{{{{\mathcal{O}}}}}}}}}(N{\eta }^{2})$ in practice^23,24^. This is a one-time additive classical cost that is not multiplied by the duration of time-evolution so it is likely subdominant to other costs.

Quantum algorithms for preparing Slater determinants have focused on the Givens rotation approach introduced in ref. ^69^ for second quantization. That algorithm requires ${{{{{{{\mathcal{O}}}}}}}}(N\eta )$ Givens rotation unitaries. Such unitaries can be implemented with ${{{{{{{\mathcal{O}}}}}}}}(\eta \log N)$ gates in first quantization^53,70^, hence combining that with the sequence of rotations called for in ref. ^69^ gives an approach to preparing Slater determinants in first quantization with $\widetilde{{{{{{{{\mathcal{O}}}}}}}}}(N{\eta }^{2})$ gates in total, a relatively high cost. Unlike the offline cost to compute the occupied orbital coefficients, this state preparation cost would be multiplied by the number of measurement repetitions.

Here, we develop a new algorithm to prepare arbitrary Slater determinants in first quantization with only $\widetilde{{{{{{{{\mathcal{O}}}}}}}}}(N\eta )$ gates. The approach is to first generate a superposition of all of the configurations of occupied orbitals in the Slater determinant while making sure that electron registers holding the label of the occupied orbitals are always sorted within each configuration so that they are in ascending order. This is necessary because, without such structure (or guarantees of something similar), the next step (anti-symmetrization) could not be reversible. For this next step, we apply the anti-symmetrization procedure introduced in ref. ^71^, which requires only ${{{{{{{\mathcal{O}}}}}}}}(\eta \log \eta \log N)$ gates (a negligible additive cost). Note that if one did not need the property that the configurations were ordered by the electron register, then it would be relatively trivial to prepare an arbitrary Slater determinant as a product state of *η* different registers, each in an arbitrary superposition over $\log N$ bits (e.g., using the brute-force state preparation of ref. ^72^).

A high-level description of how the superposition of ordered configurations comprising the Slater determinant is prepared now follows, with details given in Supplementary Note 7. The idea is to generate the Slater determinant in second quantization in an ancilla register using the Givens rotation approach of ref. ^69^, while mapping the second-quantized representation to a first-quantized representation one second-quantized qubit (orbital) at a time. One can get away with storing only *η* non-zero qubits (orbitals) at a time in the second-quantized representation because the Givens rotation algorithm gradually produces qubits that do not require further rotations. Whenever one produces a new qubit in the second-quantized representation that does not require further rotations, one can convert it to the first-quantized representation, which zeros that qubit. Thus, the procedure only requires ${{{{{{{\mathcal{O}}}}}}}}(\eta )$ ancilla qubits—a negligible additive space overhead. A total of ${{{{{{{\mathcal{O}}}}}}}}(N\eta \log N)$ gates are required because for each of ${{{{{{{\mathcal{O}}}}}}}}(N)$ steps one accesses all ${{{{{{{\mathcal{O}}}}}}}}(\eta \log N)$ qubits of the first-quantized representation. In Supplementary Note 7, we show the Toffoli complexity can be further reduced to ${{{{{{{\mathcal{O}}}}}}}}(N\eta )$ with some additional tricks.

Finally, we note that quantum algorithms can also perform finite-temperature simulation by sampling initial states from a thermal density matrix in each realization of the circuit. For example, if the system is in a regime that is well treated by mean-field theory, one can initialize the system in a Slater determinant that is sampled from the thermal Hartree–Fock state^73^. Since the output of quantum simulations already needs to be sampled this does not meaningfully increase the number of quantum repetitions required. Such an approach would also be viable classically (and would allow one to perform simulations that only ever treat *η* occupied orbitals despite having finite temperature) but would introduce a multiplicative ${{{{{{{\mathcal{O}}}}}}}}(1/{\epsilon }^{2})$ sampling cost. For either processor, there is the cost of classically computing the thermal Hartree–Fock state, but this is a one-time cost not multiplied by the duration of time-evolution or ${{{{{{{\mathcal{O}}}}}}}}(1/{\epsilon }^{2})$.

## Discussion

**Fig. 1:**
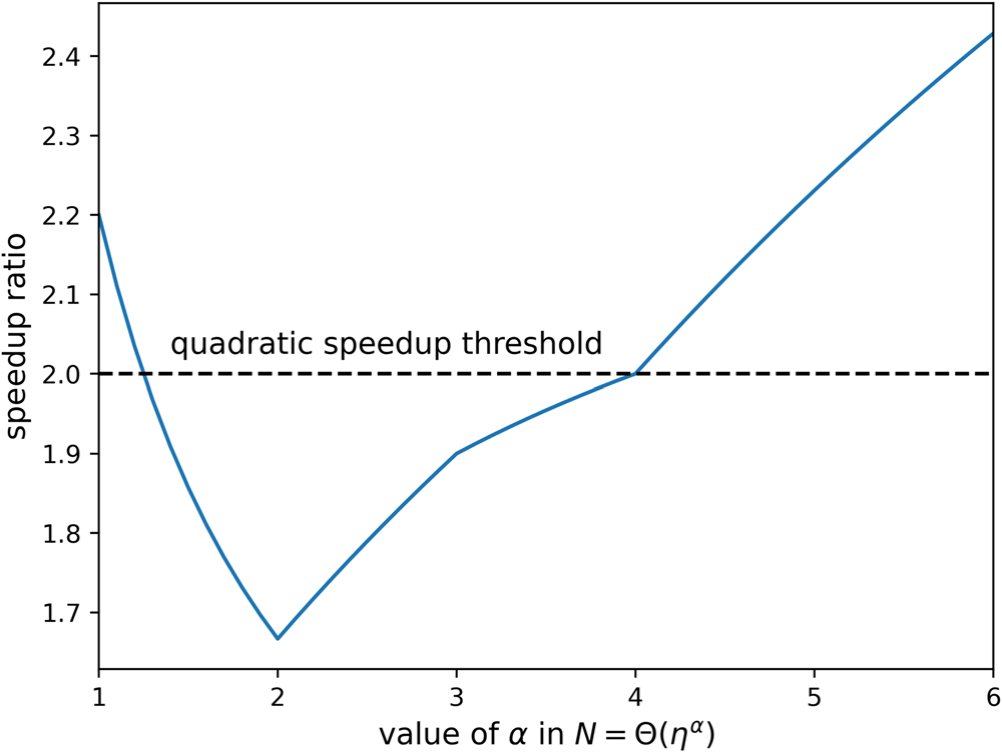
Quantum speedup ratio. Plot of the zero temperature dynamics quantum speedup ratio *β*_C_/*β*_Q_ which assumes an Θ(*η*^*α*^) relationship between problem size and basis size so that cost of the best exact quantum simulations of electron dynamics can be expressed as $({\eta }^{{\beta }_{{{{{{{{\rm{Q}}}}}}}}}}t){(Nt/\epsilon )}^{o(1)}$ and the cost of the best classical mean-field algorithms for electron dynamics can be expressed as $({\eta }^{{\beta }_{{{{{{{{\rm{C}}}}}}}}}}t){(Nt/\epsilon )}^{o(1)}$. These costs are compared under the same assumptions for sampling time-dynamics output as those mentioned in Table 1. See Supplementary Note 5 for more details.

We have reviewed and analyzed costs associated with classical mean-field methods and state-of-the-art exact quantum algorithms for electron dynamics. We tightened bounds on Trotter-based first-quantized quantum simulations and introduced new and more efficient strategies for initializing Slater determinants in first quantization and for measuring RDMs via classical shadows. We compare these costs in Table 1. We plot the speedup of quantum algorithms relative to classical mean-field approaches when the goal is to sample the output of quantum dynamics at zero temperature in Fig. 1. We see that the best quantum algorithms deliver a seventh power speedup in particle number when *N* < Θ(*η*^2^), quartic in basis size when Θ(*η*^2^) < *N* < Θ(*η*^3^), super-quadratic in basis size when Θ(*η*^3^) < *N* < Θ(*η*^4^) and quintic in basis size but with a quadratic slowdown in *η* when *N* > Θ(*η*^4^). In extremal regimes of *N* < Θ(*η*^5/4^) and *N* > Θ(*η*^4^), the overall speedup in system size is super-quadratic (see Supplementary Note 5 for details). These are large enough speedups that quantum advantage may persist even despite quantum error-correction overhead^74^. Note that our analyses are based on derivable upper bounds for both classical and quantum algorithms over all possible input states. Tighter bounds derived over restricted inputs would give asymptotically fewer time steps required for both classical and quantum Trotter algorithms^75^.

**Table 1:** Costs of exact quantum algorithms and mean-field classical algorithms for simulating fermionic dynamics

Processor	Algorithm	Observable	Space	Gate complexity
Classical	*T* = 0 mean-field with occ-RI-K/ACE^23,24^	Anything	$\widetilde{{{{{{{{\mathcal{O}}}}}}}}}(N\eta )$	$({N}^{4/3}{\eta }^{7/3}t+{N}^{5/3}{\eta }^{4/3}t){(\frac{Nt}{\epsilon })}^{o(1)}$
Classical	*T* > 0 mean-field (density matrix) with refs. ^23,24^	Anything	$\widetilde{{{{{{{{\mathcal{O}}}}}}}}}(NM)$	$({N}^{4/3}{M}^{2}{\eta }^{1/3}t\,+\,\frac{{N}^{5/3}\,{M}^{2}t}{{\eta }^{2/3}}){(\frac{Nt}{\epsilon })}^{o(1)}$
Classical	*T* > 0 mean-field (sampled trajectories) with refs. ^23,24^	Anything	$\widetilde{{{{{{{{\mathcal{O}}}}}}}}}(N\eta )$	$(\frac{{N}^{4/3}{\eta }^{7/3}t}{{\epsilon }^{2}}+\frac{{N}^{5/3}{\eta }^{4/3}t}{{\epsilon }^{2}}){(\frac{Nt}{\epsilon })}^{o(1)}$
Quantum	Second-quantized Trotter grid algorithm^45^	Sample $\left|\psi (t)\right\rangle$	${{{{{{{\mathcal{O}}}}}}}}(N\log N)$	$({N}^{4/3}{\eta }^{1/3}t+\frac{{N}^{5/3}t}{{\eta }^{2/3}}){(\frac{Nt}{\epsilon })}^{o(1)}$
Quantum	First-quantized Trotter grid algorithm here	Sample $\left|\psi (t)\right\rangle$	${{{{{{{\mathcal{O}}}}}}}}(\eta \log N)$	$({N}^{1/3}{\eta }^{7/3}t+\,{N}^{2/3}{\eta }^{4/3}t){(\frac{Nt}{\epsilon })}^{o(1)}$
Quantum	Interaction picture plane wave algorithm^51^	Sample $\left|\psi (t)\right\rangle$	${{{{{{{\mathcal{O}}}}}}}}(\eta \log N)$	$\widetilde{{{{{{{{\mathcal{O}}}}}}}}}({N}^{1/3}{\eta }^{8/3}t)$
Quantum	Grid basis algorithm from Appendix K of ref. ^53^	Sample $\left|\psi (t)\right\rangle$	${{{{{{{\mathcal{O}}}}}}}}(\eta \log N)$	$\widetilde{{{{{{{{\mathcal{O}}}}}}}}}({N}^{1/3}{\eta }^{8/3}t)$
Quantum	New shadows procedure here	*k*-RDM(*t*)	${{{{{{{\mathcal{O}}}}}}}}(\eta \log N)$	$\widetilde{{{{{{{{\mathcal{O}}}}}}}}}({k}^{k}{\eta }^{k}L\,{{{{{{{{\mathcal{C}}}}}}}}}_{{{{{{{{\rm{samp}}}}}}}}}/{\epsilon }^{2})$
Quantum	Gradient measurement^61^	$\left\langle \psi (t)\right|O\left|\psi (t)\right\rangle$	$\widetilde{{{{{{{{\mathcal{O}}}}}}}}}(\eta+L)$	$\widetilde{{{{{{{{\mathcal{O}}}}}}}}}(\sqrt{L}\,{{{{{{{{\mathcal{C}}}}}}}}}_{{{{{{{{\rm{samp}}}}}}}}}\,\lambda /\epsilon )$
Quantum	Gradient measurement^61^	$\left\langle \psi (t)\right|H\left|\psi (t)\right\rangle$	$\widetilde{{{{{{{{\mathcal{O}}}}}}}}}(\eta+L)$	$\widetilde{{{{{{{{\mathcal{O}}}}}}}}}(\frac{\sqrt{L}{{{{{{{{\mathcal{C}}}}}}}}}_{{{{{{{{\rm{samp}}}}}}}}}t({N}^{1/3}{\eta }^{5/3}\,+{N}^{2/3}{\eta }^{1/3})}{\epsilon })$

*N* is the number of basis functions, *η* is the number of particles, *ϵ* is target precision, *M* is the number of appreciably occupied orbitals in a finite-temperature (*T*) simulation (*M* ≃ *N* for high *T*), *O* is any observable having norm *λ* that can be block encoded with cost less than time-evolution, *t* is the duration of evolution, *L* is the number of time points at which we wish to resolve quantities and ${{{{{{{{\mathcal{C}}}}}}}}}_{{{{{{{{\rm{samp}}}}}}}}}$ is the cost of sampling $\left|\psi (t)\right\rangle$ with a quantum algorithm. We are not accounting for the additive time-independent costs of state preparation ($\widetilde{{{{{{{{\mathcal{O}}}}}}}}}(\eta N)$ gates using the procedure of Supplementary Note 7) or of classically reconstructing the *k*-RDM given measurement outcomes. Thus, this table reports gate complexities for long-time *t* simulations. In Supplementary Note 5 we provide a table clarifying which algorithm has optimal gate complexity as a function of *N*/*η*.

The story becomes more nuanced when we wish to estimate *ϵ*-accurate quantities via sampling the quantum simulation output at *L* different time points. For observables with norm scaling as ${{{{{{{\mathcal{O}}}}}}}}(1)$ (e.g., simple correlation functions or single RDM elements), or those pertaining to amplitudes of the state (e.g., scattering amplitudes or reaction rates), the scaling advantages in system and basis size are maintained but at the cost of the quantum algorithm slowing down by a multiplicative factor of at least ${{{{{{{\mathcal{O}}}}}}}}(\sqrt{L}/\epsilon )$. When targeting the 1-RDM (which characterizes all observables within mean-field theory) we maintain speedup in *N* but at the cost of an additional linear slowdown in *η*. When measuring the total energy, the overall speedup becomes tenuous. Thus, the viability of quantum advantage with respect to zero-temperature classical mean-field methods depends sensitively on the target precision and particular observables of interest.

In terms of applications, we expect RT-TDHF to provide qualitatively correct dynamics whenever electron correlation effects are not pronounced. RT-TDDFT includes some aspects of electron correlation but the adiabatic approximation often creates issues^76^ and the method suffers from self-interaction error^77^. When the adiabatic approximation is accurate, self-interaction error is not pronounced, and the system does not exhibit a strong correlation, we expect RT-TDDFT to generate qualitatively correct dynamics. When there are many excited states to consider for spectral properties, it is often beneficial to resort to real-time dynamics methods instead of linear-response methods. Furthermore, we are often interested in real-time non-equilibrium electronic dynamics. This is the case for photo-excited molecules near metal surfaces^78^. The time evolution of electron density (i.e., the diagonal of the 1-RDM) near the molecule is of particular interest due to its implications for chemical reactivity and kinetics in the context of heterogeneous catalysis^79^. In this application, the simulation of nuclear degrees of freedom may be equally important, which we will leave for future analysis.

We see from Table 1 that prospects for quantum advantage are considerably increased at finite temperatures. Thus, a promising class of problems to consider for speedup over mean-field methods is the electronic dynamics of either warm dense matter (WDM)^80–83^ or hot dense matter (HDM)^84^. The WDM regime (where thermal energy is comparable to the Fermi energy) is typified by temperatures and densities that require the accurate treatment of both quantum and thermal effects^85,86^. These conditions occur in planetary interiors, experiments involving high-intensity lasers, and inertial confinement fusion experiments as the ablator and fuel are compressed into the conditions necessary for thermonuclear ignition. Ignition occurs in the hot dense matter (HDM) regime (where thermal energy far exceeds the Fermi energy). While certain aspects of these systems are conspicuously classical, they represent a regime that can be challenging to model, particularly the opacity of matter in stellar atmospheres^87,88^. Such astrophysical applications often require spectroscopic accuracy which is orders of magnitude more precise than chemical accuracy and necessitates a high ratio of *N* to *η* (the regime where the speedup of quantum algorithms relative to classical mean-field is most pronounced).

Another interesting context arises due to the conditions in which WDM is created in a laboratory. High-intensity ultrafast lasers or charged particle beams incident on condensed phase samples can be used to create these conditions on femtosecond time scales and the associated strong excitation and subsequent relaxation are well beyond the capabilities of mean-field methods^89^. Classical algorithms for HDM typically rely on an average atom description of the system in which the entire electronic structure is reduced to that of a single atom self-consistently embedded in a plasma^90–93^. While the level of theory applied to this atom can be sophisticated, the larger-scale structure of such a plasma is treated at a mean-field level. Identifying suitable observables for both of these regimes remains ongoing work.

Simulations in either the WDM or HDM regime typically rely on large plane wave basis sets and the inclusion of 10–100 times more partially occupied orbitals per atom than would be required at lower temperatures. Often, the attendant costs are so great that it is impractical to implement RT-TDDFT with hybrid functionals. Therefore, many calculations necessarily use adiabatic semi-local approximations, even on large classical high-performance computing systems^80^. Thus, the level of practically achievable accuracy can be quite low, and the prospect of exactly simulating the dynamics on a quantum computer is particularly compelling.

Although we have focused on assessing quantum speedup over mean-field theory, we view the main contribution of this work as more general. In particular, if exact quantum simulations are sometimes more efficient than classical mean-field methods, then all levels of theory in between mean-field and exact diagonalization are in scope for possible quantum advantage. Targeting systems that require more correlated calculations narrows the application space but improves prospects for quantum advantage due to the unfavorable scaling of the requisite classical algorithms. Thus, it may turn out that the domain of systems requiring, say, coupled cluster dynamics^94–97^, might be an even more ideal regime for practical quantum advantage, striking a balance in the trade-off between the breadth of possible applications and the cost of the classical competition.

## Supplementary information

Supplementary Information

Peer Review

## Data Availability

The authors declare that the data supporting the findings of this study are available within the paper.

## Code availability

The source code for generating Fig. 1 is available from the corresponding authors upon request.
