# Supplementary material for: Quantum simulation of exact electron dynamics can be more efficient than classical mean-field methods
Source: Nat Commun. 2023 Jul 10;14:4058. doi: 10.1038/s41467-023-39024-0 (PMC10333266; doi:10.1038/s41467-023-39024-0)
Supplement: Supplementary file 1 — Supplementary Information [file 41467_2023_39024_MOESM1_ESM.pdf]

Supplementary Information for "Quantum simulation of exact electron dynamics can be more efficient than classical mean-field methods"

Ryan Babbush[1,*], William J. Huggins[1], Dominic W. Berry[2], Shu Fay Ung[3], Andrew Zhao[1,4], David R. Reichman[3], Hartmut Neven[1], Andrew D. Baczewski[5], and Joonho Lee[1,3,**]

[1]Google Quantum AI, Venice, CA, United States
[2]Department of Physics and Astronomy, Macquarie University, Sydney, NSW, Australia
[3]Department of Chemistry, Columbia University, New York, NY, United States
[4]Department of Physics and Astronomy, University of New Mexico, Albuquerque, NM, United States
[5]Quantum Algorithms and Applications Collaboratory, Sandia National Laboratories, Albuquerque NM, United States
[*]Corresponding author: babbush@google.com
[**]Corresponding author: joonholee@g.harvard.edu

Contents

1 Norms and scaling for the nonlinear differential equation governing mean-field evolution **3**

2 Proving sublinear gate complexity in basis size for Trotter based methods **8**

3 Constant factors for time-evolution in the interaction-picture plane-wave algorithm **11**

4 Smoothing the Coulomb operator exponentially suppresses quantum scaling in basis size **12**

5 Gate complexity and speedup in various regimes **14**

6 Efficient reduced density matrix estimation using classical shadows in first quantization **17**

6.1 Problem statement 17

6.2 The measurement protocol 19

6.3 Notation and preliminaries 23

6.4 Variance of the k-RDM with a restricted sum 27

6.4.1 Removing the off-diagonal terms 29

6.4.2 Taking advantage of antisymmetry 31

6.4.3 Putting the pieces together 34

7 More efficient Slater determinant state preparation in first quantization **38**

Supplementary Note 1 Norms and scaling for the nonlinear differential equation governing mean-field evolution

We have the differential equation

$$i\frac{\partial \mathbf{C}_{\text{occ}}(t)}{\partial t} = \mathbf{F}(t)\,\mathbf{C}_{\text{occ}}(t) \tag{1}$$

where

$$F_{\mu\nu}(t) = h_{\mu\nu} + \sum_{\lambda\sigma}^{N}\left((\mu\nu|\lambda\sigma) - \frac{(\mu\sigma|\lambda\nu)}{2}\right)P_{\sigma\lambda}(t) \tag{2}$$

with $\mathbf{P}(t) = \mathbf{C}_{\text{occ}}(t)\mathbf{C}_{\text{occ}}(t)^{\dagger}$. If $\mathbf{F}$ were independent of $\mathbf{C}_{\text{occ}}$, then it would imply that taking the nth derivative gives

$$(i)^n\frac{\partial^n \mathbf{C}_{\text{occ}}(t)}{\partial t^n} = \mathbf{F}^n\mathbf{C}_{\text{occ}}(t)\,. \tag{3}$$

That means the norm of the nth derivative would scale as $\|\mathbf{F}\|^n$ (with $\mathbf{C}_{\text{occ}}$ normalized).

Then higher-order methods will typically have an error that scales as the norm of the higher-order derivatives. For example, if one were to use a Taylor series up to order k to approximate a time step, then the error for a time step of length δt would scale as

$$\frac{1}{(k+1)!}\|\mathbf{F}\|^{k+1}\delta t^{k+1}. \tag{4}$$

This means that if the size of the time step is taken as proportional to $1/\|\mathbf{F}\|$, then the error may be made exponentially small in k. As a result, the total number of time steps used scales as $\mathcal{O}(\|\mathbf{F}\|t)$. Similar considerations hold for other higher-order methods for integration. The dependence of the complexity on $\|\mathbf{F}\|$ can also be expected from principles of scaling, where if $\mathbf{F}$ is divided by $\|\mathbf{F}\|$ but t is also multiplied by $\|\mathbf{F}\|$, then the same differential equation is obtained.

In our case where $\mathbf{F}$ is dependent on $\mathbf{C}_{\text{occ}}$, the situation is more complicated. This is because taking higher-order derivatives of $\mathbf{C}_{\text{occ}}$ yields more terms due to the derivatives of $\mathbf{C}_{\text{occ}}$ in $\mathbf{F}$. To

describe this, let us write, omitting $h_{\mu\nu}$ for simplicity,

$$F_{\mu\nu}(t) = V_{\mu\nu\sigma\lambda} C_{\sigma a} C^*_{\lambda a}, \tag{5}$$

with $C_{\sigma a}$ the matrix entries of $\mathbf{C}_{\text{occ}}$. We are taking a convention that Greek indices are over all orbitals, English letters are over electrons, and repeated indices are summed over. Then we would give the derivative as

$$i\frac{\partial C_{\mu b}}{\partial t} = V_{\mu\nu\sigma\lambda} C_{\sigma a} C^*_{\lambda a} C_{\nu b}. \tag{6}$$

We can define an η-norm of the discretized potential operator $\mathbf{V}$ as

$$\|\mathbf{V}\|_\eta = \max_{\mathbf{x},\mathbf{y},\mathbf{z}} V_{\mu\nu\sigma\lambda} x_\mu y^*_\nu z_{\sigma\lambda}, \tag{7}$$

with $\|\mathbf{x}\| = \|\mathbf{y}\| = \|\mathbf{z}\| = 1$ (i.e., spectral norms are normalized), and $\mathbf{z}$ of rank η. To bound this norm, we can consider the first term for $V_{\mu\nu\sigma\lambda}$, which is

$$(\mu\nu|\lambda\sigma) = \int \mathrm{d}\mathbf{r}_1\, \mathrm{d}\mathbf{r}_2 \frac{\phi^*_\mu(\mathbf{r}_1)\,\phi_\nu(\mathbf{r}_1)\,\phi^*_\lambda(\mathbf{r}_2)\,\phi_\sigma(\mathbf{r}_2)}{|\mathbf{r}_1 - \mathbf{r}_2|}\,. \tag{8}$$

The multiplication by x_μ and sum over μ corresponds to a transformation of ϕ_μ to a new orbital, and similarly, the sum over ν transforms ϕ_ν to another new orbital. Since $\mathbf{z}$ is of rank η, the sum over λ and σ corresponds to transforming the orbital basis for both ϕ_λ and ϕ_σ, and summing over η of these basis states.

That is, we can write

$$\sum_{a=1}^{\eta} \int \mathrm{d}\mathbf{r}_1\, \mathrm{d}\mathbf{r}_2 \frac{\phi^*(\mathbf{r}_1)\,\chi(\mathbf{r}_1)\,\psi^*_a(\mathbf{r}_2)\,\theta_a(\mathbf{r}_2)}{|\mathbf{r}_1 - \mathbf{r}_2|}\,, \tag{9}$$

for some transformed orbitals $\phi, \chi, \psi_a, \theta_a$. We can then use the fact that $|\phi^*\chi| \le |\phi|^2 + |\chi|^2$, and similarly for ψ_a and θ_a to upper bound this expression by

$$4\sum_{a=1}^{\eta} \int \mathrm{d}\mathbf{r}_1\, \mathrm{d}\mathbf{r}_2 \frac{|\phi(\mathbf{r}_1)|^2 |\psi_a(\mathbf{r}_2)|^2}{|\mathbf{r}_1 - \mathbf{r}_2|}\,, \tag{10}$$

for some choice of ϕ and ψ_a. This integral can be maximized when the ψ_a are orbitals that are clustered as close as possible to ϕ. With neighboring grid points separated by δ, the smallest average separation can be is $\mathcal{O}(\eta^{1/3}\delta)$. Then the factor of $1/|\mathbf{r}_1 - \mathbf{r}_2|$ in the integrals will give $\mathcal{O}(1/[\eta^{1/3}\delta])$. Multiplying the sum by η gives $\mathcal{O}(\eta^{2/3}/\delta)$.

The second term for $V_{\mu\nu\sigma\lambda}$ is $(\mu\sigma|\lambda\nu)$. This is similar to $(\mu\nu|\lambda\sigma)$, but with ν and σ swapped. Then the transformation of orbitals gives

$$\sum_{a=1}^{\eta} \int \mathbf{d}\mathbf{r}_1 \, \mathbf{d}\mathbf{r}_2 \frac{\phi^*(\mathbf{r}_1)\,\chi_a(\mathbf{r}_1)\,\psi_a^*(\mathbf{r}_2)\,\theta(\mathbf{r}_2)}{|\mathbf{r}_1 - \mathbf{r}_2|}\,, \tag{11}$$

for some choice of $\phi, \chi_a, \psi_a, \theta$. The same argument holds, where the sum is maximized with orbitals over a region of volume $\eta\delta^3$ so there are contributions from all η terms in the sum, but $1/|\mathbf{r}_1 - \mathbf{r}_2|$ averages to give $\mathcal{O}(1/[\eta^{1/3}\delta])$. This gives the same scaling for the second term for $V_{\mu\nu\sigma\lambda}$, and so

$$\|\mathbf{V}\|_\eta = \mathcal{O}(\eta^{2/3}/\delta). \tag{12}$$

What this means is that, whenever we have a contraction of the σ, λ indices in $V_{\mu\nu\sigma\lambda}$ with a normalized matrix of rank η, the remaining matrix has norm $\mathcal{O}(\eta^{2/3}/\delta)$. That immediately implies that $\|\mathbf{F}\|$ has this norm. Then applying $\mathbf{F}$ to the normalized matrix $\mathbf{C}_{\rm occ}$ gives an upper bound on the first derivative $\mathcal{O}(\eta^{2/3}/\delta)$. A similar formal analysis can be found in [1].

Taking the second derivative then yields an expression with 3 terms, where each has $\mathbf{V}$ appearing twice and $\mathbf{C}_{\rm occ}$ appearing five times. In particular,

$$\begin{aligned}
-\frac{\partial^2 C_{\mu b}}{\partial t^2} &= V_{\mu\nu\sigma\lambda}[(V_{\sigma\epsilon\zeta\eta}C_{\zeta c}C^*_{\eta c})C_{\epsilon a}C^*_{\lambda a}]C_{\nu b} \\
&\quad + V_{\mu\nu\sigma\lambda}[C_{\sigma a}(V_{\lambda\epsilon\zeta\eta}C^*_{\zeta c}C_{\eta c})C^*_{\epsilon a}]C_{\nu b} \\
&\quad + (V_{\mu\nu\sigma\lambda}C_{\sigma a}C^*_{\lambda a})(V_{\nu\epsilon\zeta\eta}C_{\zeta c}C^*_{\eta c})C_{\epsilon b}
\end{aligned} \tag{13}$$

Only the third line has a simple interpretation as $\mathbf{F}$ squared times $\mathbf{C}_{\rm occ}$ (indicated by the brackets).

The first line has $\mathbf{V}$ contracted with $\mathbf{C}_{\rm occ}$ using ζ, η, so the expression in round brackets is a matrix with norm $\mathcal{O}(\eta^{2/3}/\delta)$. Then in matrix terms, it is multiplied by $C_{\epsilon a}C^*_{\lambda a}$ (summed over a), which is a matrix of norm 1 and rank η. As a result, the expression in square brackets is of norm $\mathcal{O}(\eta^{2/3}/\delta)$ and rank η. We can then see that the first $\mathbf{V}$ is contracted over σ, λ with a matrix of rank η and norm $\mathcal{O}(\eta^{2/3}/\delta)$. That implies that the norm of the resulting matrix is upper bounded by the square of $\mathcal{O}(\eta^{2/3}/\delta)$. That is then multiplied by $C_{\nu b}$ which is of norm 1, resulting in the overall norm of this line being upper bounded by the square of $\mathcal{O}(\eta^{2/3}/\delta)$. Similar considerations hold for the second line, so we can upper bound the entire second derivative by an order scaling that is the square of that for $\|\mathbf{F}\|$.

In this, the general principle is that wherever we have something of the form $C_{\sigma a}C^*_{\lambda a}$, it is a matrix of norm 1 and rank η, and taking the derivative of it yields something that is still of rank η, but with a norm upper bounded by $\mathcal{O}(\eta^{2/3}/\delta)$. Because we have bounded the norm when contracting $\mathbf{V}$ with a general matrix of rank η, that yields a factor of $\mathcal{O}(\eta^{2/3}/\delta)$ on whatever result we had for the lower-order derivative. The other scenario is where we take the derivative of $C_{\nu b}$, which is effectively like multiplying it by $\mathbf{F}$ which increases the norm (but not the rank).

This reasoning holds in general whenever we take the derivative of an expression for the derivative of some order to give the derivative of higher order. The norm is multiplied by $\mathcal{O}(\eta^{2/3}/\delta)$ for each of the terms. The number of terms will increase exponentially with the order. The third derivative has 3×5 terms, where each of the three original terms yields five due to the derivatives of $\mathbf{C}_{\rm occ}$ at each location. Then the fourth-order derivative has $3 \times 5 \times 7$ terms and so on. In describing the scaling we can ignore this exponential number of terms, and give the upper bound on the nth order derivative as $\mathcal{O}(\eta^{2/3}/\delta)$ to the power of n. This implies that the appropriate scaling of the time should again be $T = \|\mathbf{F}\|t$.

Finally we bound the norm of $\|\mathbf{h}\|$. When using a plane wave basis, $h_{\mu\nu}$ will be non-zero only

when $\mu = \nu$ with entries scaling as $\mathcal{O}(1/\delta^2)$ due to the ∇^2 in the expression for $h_{\mu\nu}$. That gives the scaling of the spectral norm for this component, which would be unchanged under a unitary transformation, such as the Fourier transform which maps plane waves to an approximately local basis.

For the dependence of $h_{\mu\nu}$ on $V(r)$, the potential will come from nuclei, and for charge-neutral systems the total nuclear charge will be the same as the number of electrons. If the nuclear charge were entirely at one location and we have a charge-neutral system, then the largest contribution to $h_{\mu\nu}$ would be for an approximately local basis, where the contribution would scale as η/δ, with the factor of η from the nuclear charge and $1/\delta$ from the inverse distance.

In most cases that we would be interested in, there would be a more even distribution of nuclear charges through the volume. In that case, if the volume scales as η, there would be an average distance $\mathcal{O}(\eta^{1/3})$. That would result in a contribution to $h_{\mu\nu}$ of $\mathcal{O}(\eta^{2/3})$. An orbital localized near one nucleus would give a contribution of $\mathcal{O}(1/\delta)$ just from that nucleus, which may be larger than $\mathcal{O}(\eta^{2/3})$ if $N > \eta^3$ but may be ignored in comparison to $1/\delta^2$.

As a result of these considerations, we can give the upper bound on $\mathbf{F}$ in the case without $V(r)$ as

$$\|\mathbf{F}\| = \mathcal{O}\left(\frac{\eta^{2/3}}{\delta} + \frac{1}{\delta^2}\right). \tag{14}$$

In the case with nuclei we obtain the same result, provided the nuclear charges are not clustered any closer than the grid spacing. Here $\delta = \mathcal{O}((\eta/N)^{1/3})$ is the minimum grid spacing. This scaling for δ comes from taking the computational cell volume proportional to η (a reasonable assumption for both condensed-phase and molecular systems). Thus, the scaling becomes

$$\|\mathbf{F}\| = \mathcal{O}\left(N^{1/3}\eta^{1/3} + \frac{N^{2/3}}{\eta^{2/3}}\right). \tag{15}$$

In this case we can see that the first term is dominant unless $N > \eta^3$.

Supplementary Note 2 Proving sublinear gate complexity in basis size for Trotter based methods

Here we derive the complexity for quantum simulation of the electronic structure problem given in Eq. (12). We consider the simulation of the electronic structure problem defined on a spatial grid in first quantization. Note that the problem of sampling from the output of the quantum dynamics after some time will be specified completely by the following inputs: (1) the locations R_ℓ and charges ζ_ℓ of the L nuclei, (2) the duration of time-evolution t, (3) the number of electrons η, (4) the number of basis functions N, (5) the target precision to within which one realizes the correct unitary ϵ and (6) the initial state. The Hamiltonian whose simulation we target can be expressed as

$$H = T + U + V + \sum_{\ell \neq \kappa = 1}^{L} \frac{\zeta_\ell \zeta_\kappa}{2 \left\| R_\ell - R_\kappa \right\|} \tag{16}$$

$$T \approx \sum_{i=1}^{\eta} \mathrm{QFT}_j \left(\sum_{p \in G} \frac{\left\| k_p \right\|^2}{2} |p\rangle\langle p|_j \right) \mathrm{QFT}_j^\dagger \tag{17}$$

$$U = -\sum_{j=1}^{\eta} \sum_{\ell=1}^{L} \sum_{p \in G} \frac{\zeta_\ell}{\left\| R_\ell - r_p \right\|} |p\rangle\langle p|_j \tag{18}$$

$$V = \sum_{j \neq k=1}^{\eta} \sum_{p,q \in G} \frac{1}{2 \left\| r_p - r_q \right\|} |p\rangle\langle p|_j \, |q\rangle\langle q|_k \tag{19}$$

where QFT_j is the usual quantum Fourier transform applied to register j. We emphasize that T is only approximately given by the expression involving the QFT. This relation is exact in the continuum limit where $N \to \infty$. For finite-sized grids N, it cannot be the case that the QFT completely diagonalizes the momentum operator. Instead, writing T this way represents something similar to the approximations made by so-called "discrete value representation" methods. Using the QFT means that the evolution can be broken into a product of the evolution under T and the one under $U + V$.

In the above expression, ℓ and κ index nuclear degrees of freedom and we have the following

definition of grid points and their frequencies in the dual space defined by the QFT:

$$r_p = \frac{p\,\Omega^{1/3}}{N^{1/3}} \qquad k_p = \frac{2\pi p}{\Omega^{1/3}} \qquad p \in G \qquad G = \left[-\frac{N^{1/3}-1}{2}, \frac{N^{1/3}-1}{2}\right]^3 \subset \mathbb{Z}^3\,, \tag{20}$$

where Ω is the volume of the simulation cell and N is the number of grid points in the cell. Although it is defined here in more precise terms, this is essentially the same representation used in the first work on quantum simulating chemistry in first quantization, by Kassal *et al.* [2], well over a decade ago. The Trotter errors associated with this grid representation were also investigated numerically recently by Chan *et al.* [3].

We consider simulation performed using high-order product formulas with a split-operator Trotter step. What we mean by the latter is that we will alternate evolution under T (using the QFT) and evolution under $U + V$. In fact, the implementation of each Trotter step that we will pursue is essentially identical to the Trotter steps proposed by Kassal *et al.* [2]. The Trotter step requires $\widetilde{\mathcal{O}}(\eta^2)$ gates, with the complexity being dominated by computing the $\mathcal{O}(\eta^2)$ different interactions in the two-electron term. We remark that some of the constant factors associated with this computation were worked out over a decade ago by Jones *et al.* [4]. Recently, Low *et al.* [1] have shown that the number of Trotter steps required in second quantization using arbitrarily high order formulas can be as low as

$$\left(N^{1/3}\eta^{1/3} + \frac{N^{2/3}}{\eta^{2/3}}\right)\frac{t^{1+o(1)}N^{o(1)}}{\epsilon^{o(1)}}\,. \tag{21}$$

We note that, curiously, this also closely matches our bound for the norm of the Fock operator (see Eq. (15)) proved in Supplementary Note 1. The first term in brackets similarly corresponds to a contribution to the potential from electrons grouped as closely as possible in real space, but the reason why this quantity is relevant is very different between the two calculations.

The results for the Trotter error in second quantization also hold for first quantization. As a general principle, we can consider the effect of $\sum_j |p\rangle\langle q|_j$ on a computational basis state

consisting of an anti-symmetric combination of lists of electron positions. This removes an electron from orbital q and places it in p. This is performed for every part of the anti-symmetric state, preserving its sign. However, for the starting anti-symmetric state the sign is based on whether the permutation is even or odd (as compared to ascending order). If moving an electron from q to p passes over an *odd* number of electrons, then the parity of each permutation flips. That means that there is an overall sign flip in the basis state.

Similarly, if we consider the action of $a_p^\dagger a_q$ on a state $a_{q_1}^\dagger \cdots a_{q_\eta}^\dagger |0\rangle$, then the a_q can be anti-commuted to the right to give several sign flips corresponding to the number of $a_{q_j}^\dagger$ operators that are anti-commuted through. This corresponds to the number of occupied orbitals before q. Then $a_q a_q^\dagger$ gives the identity. Next, anti-commute $a_p^\dagger$ to the appropriate location in the list of operators. The sign that is obtained corresponds to the number of $a_{q_j}^\dagger$ operators that are anti-commuted through, which is the number of electrons before p. There is an overall sign flip if there is an odd number of electrons between p and q.

This can then be extended to products such as

$$\sum_j |p\rangle \langle q|_j \sum_k |r\rangle \langle s|_k \,. \tag{22}$$

The first sum corresponds to $a_p^\dagger a_q$ in second quantization, and the second sum corresponds to $a_r^\dagger a_s$. This means that we have the equivalence

$$\sum_{pqrs} V_{pqrs} \sum_j |p\rangle \langle q|_j \sum_k |r\rangle \langle s|_k \equiv \sum_{pqrs} V_{pqrs} a_p^\dagger a_q a_r^\dagger a_s. \tag{23}$$

The action on an anti-symmetric computational basis state in first quantization has exactly the same effects as that on the corresponding second-quantization state with η electrons. Moreover, the action of the operators always preserves the electron number in second quantization, so there is a corresponding state in first quantization. Similarly, because we are using anti-symmetric states in first quantization, it is impossible to obtain a state with multiple electrons on the same

orbital. That is because two registers with the same orbital number will give cancellation of terms.

As a result all operators and states in second-quantization map directly to first quantization, preserving the norms, and in particular the error bounds derived in second-quantization hold for first quantization. Therefore, multiplying the number of steps in Eq. (21) by the $\widetilde{\mathcal{O}}(\eta^2)$ gate complexity required of the first-quantized Trotter step from [2] gives the following gate complexity for the product formula based time evolution in first quantization:

$$\left(N^{1/3}\eta^{7/3} + N^{2/3}\eta^{4/3}\right) \frac{t^{1+o(1)} N^{o(1)}}{\epsilon^{o(1)}} \,. \tag{24}$$

This is the complexity given in Eq. (12).

Supplementary Note 3 Constant factors for time-evolution in the interaction-picture plane-wave algorithm

Here we analyze the constant factors in the scaling of the interaction picture based plane wave algorithm from Babbush *at al.* [5] which was analyzed in detail for use in phase estimation by Su *et al.* [6]. As explained on page 30 of [6], the number of steps to give total time T using the time evolution approach is $\lambda_B T/\ln 2$, but with a factor of 3 overhead for amplitude amplification. Using the qubitization approach the number of steps is $e\lambda_B T$. That means simulating the time evolution gives an overhead of $3/(e\ln 2) \approx 1.59$ over the qubitization. Then in Eq. (154) of [6], the total time of evolution is approximately $\pi/(2\epsilon_{\rm pha})$ to give precision $\epsilon_{\rm pha}$ of the phase estimation. There is moreover a (small) term $\mathcal{O}((\lambda_U + \lambda_V)^2 \Delta E^2)$ in the expression for the number of steps $\mathcal{N}$ in [6] that originates from the nonlinearity of the sine function in phase estimation, which is not used here.

As a result, the complexity given in Theorem 5 of [6] can be modified to be appropriate for time

evolution simply by replacing the formula for the number of steps in Eq. (174) of [6] with

$$\mathcal{N} = \frac{3T(\lambda_U^1 + \lambda_V^1/(1-1/\eta))}{P_{\rm eq}\ln 2} + \mathcal{O}(1)\,. \tag{25}$$

Here we have replaced $\pi/(2\epsilon_{\rm pha})$ with T, replaced e with $e/\ln 2$, and removed $\mathcal{O}((\lambda_U + \lambda_V)^2\Delta E^2)$. Note that in this expression

$$\lambda_U = \frac{\eta\sum_\ell \zeta_\ell}{\pi\Omega^{1/3}}\lambda_\nu, \tag{26}$$

$$\lambda_V = \frac{\eta(\eta-1)}{2\pi\Omega^{1/3}}\lambda_\nu, \tag{27}$$

$$\lambda_\nu = \sum_{\nu\in G_0}\frac{1}{\|\nu\|^2} \le 4\pi N^{1/3}, \tag{28}$$

$\lambda_U^1 \approx \lambda_U$, $\lambda_V^1 \approx \lambda_V$, and $P_{\rm eq}$ is close to 1. This expression together with an appropriate choice of constant factor in $\Omega \propto \eta$ gives the constant factor for the number of steps to use for time evolution. It needs to be multiplied by a further complicated expression in Theorem 5 of [6] for the gate complexity of a single step to provide the full constant factor for the gate complexity in Eq. (13).

Supplementary Note 4 Smoothing the Coulomb operator exponentially suppresses quantum scaling in basis size

Here we discuss the fact that if one is willing to introduce a slight systematic bias into the Coulomb operator, it is possible to further improve the speedup in N of the quantum algorithm. The $N^{1/3}$ dependence enters into the cost from the 1-norm of the two-body Coulomb operator, which scales as $\lambda = \mathcal{O}(\eta^2 V_{\rm max})$ where $V_{\rm max}$ is the maximum value of the electron-electron interaction for a single pair of electrons.

For typical plane wave or grid discretizations we have that $V_{\rm max} = \mathcal{O}(N^{1/3}/\Omega^{1/3})$ where Ω is the size of the computational cell (for the purpose of the analysis in this paper we assume that

$\Omega = \mathcal{O}(\eta)$, since that is explicitly the case in condensed phase simulations). But we could also take steps to smooth out the cusp in the Coulomb operator and thus, lower the energy scale of $V_{\max}$. For example, this could be accomplished by taking $V_{\max}$ to be a constant and modifying the real-space form of the two-body Coulomb operator as

$$\frac{1}{|r_1 - r_2|} \to \frac{1}{|r_1 - r_2| + V_{\max}} \,. \tag{29}$$

Such a strategy has been explored in the context of first-quantized quantum algorithms in real space in papers by Kivlichan *et al.* [7] and Childs *et al.* [8].

In principle, one could choose $V_{\max} = \mathcal{O}(\log N)$ and this would lead to the quantum algorithm scaling exponentially better than classical algorithms in N. This would also slightly reduce the cost of classical mean-field algorithms from scaling as $N^{4/3}$ to scaling as N. Of course, using such a drastic cutoff will introduce a significant bias into the overall dynamics. In order to avoid this, papers such as [9, 10] have sought to develop Richardson extrapolation type schemes where simulations are run with a series of smoothing or cutoff parameters in order to extrapolate the value of the observable with zero cutoff. However, questions remain about the convergence of such procedures and it seems likely to re-introduce some polynomial dependence on N in order to reach convergence with the continuum limit.

Nevertheless, the context of this paper is that one might be interested in getting a speedup over low accuracy classical algorithms. In that spirit, one could probably make the case that if merely trying to improve in speed over mean-field algorithms, the error introduced in imposing a cutoff in the Coulomb operator might be less significant than the error due to making the mean-field approximation. Thus, this is perhaps a valid approach when competing with such classical methods, and thus might provide an exponential speedup.

Supplementary Note 5 Gate complexity and speedup in various regimes

Another way to express the results of Table 1 is as a formula for the leading order scaling if assume that $N = \Theta(\eta^\alpha)$. Then, for the classical algorithm we have that the leading gate complexity of the best approach is

$$\left(\eta^\beta t\right)\left(\frac{Nt}{\epsilon}\right)^{o(1)} \qquad \text{where} \qquad N = \Theta\left(\eta^\alpha\right) \qquad \text{and} \qquad \beta = \begin{cases} \frac{4\alpha+7}{3} & \alpha \leq 3 \\ \frac{5\alpha+4}{3} & \alpha \geq 3 \end{cases}. \tag{30}$$

By contrast, for the quantum algorithm we have that the leading order gate complexity of the best approach is

$$\left(\eta^\beta t\right)\left(\frac{Nt}{\epsilon}\right)^{o(1)} \qquad \text{where} \qquad N = \Theta\left(\eta^\alpha\right) \qquad \text{and} \qquad \beta = \begin{cases} \frac{4\alpha+1}{3} & \alpha \leq 2 \\ \frac{\alpha+7}{3} & 2 \leq \alpha \leq 3 \\ \frac{2\alpha+4}{3} & 3 \leq \alpha \leq 4 \\ \frac{\alpha+8}{3} & \alpha \geq 4 \end{cases}. \tag{31}$$

For both classical and quantum expressions, these complexities are sometimes loose by sub-polynomial factors. Finally, we compare the speedup that exact quantum algorithms offer over classical mean-field algorithms. We report this as

$$\frac{\text{exponent of } \eta \text{ scaling of classical complexity}}{\text{exponent of } \eta \text{ scaling of quantum complexity}} = \begin{cases} \left(4\alpha+7\right)/\left(4\alpha+1\right) & \alpha \leq 2 \\ \left(4\alpha+7\right)/\left(\alpha+7\right) & 2 \leq \alpha \leq 3 \\ \left(5\alpha+4\right)/\left(2\alpha+4\right) & 3 \leq \alpha \leq 4 \\ \left(5\alpha+4\right)/\left(\alpha+8\right) & \alpha \geq 4, \end{cases} \tag{32}$$

if $N = \Theta\left(\eta^\alpha\right)$. We plot numerical values of this speedup in the main text in Figure 1.

Finally, we discuss the hope that Trotter based first-quantized algorithms might be sped up by a factor of $\widetilde{\mathcal{O}}(\eta)$ by developing more efficient Trotter steps. The bottleneck for Trotter steps is the computation of the Coulomb operator since the simulation of the kinetic operator scales as $\widetilde{\mathcal{O}}(\eta)$. Thus, it seems promising that fast-multipole [13] Barnes-Hut [14], or particle-mesh Ewald [15] type algorithms for computing the Coulomb potential require $\widetilde{\mathcal{O}}(\eta)$ operations in the

Processor	Algorithm for sampling $\lvert\psi(t)\rangle$	Regime of optimality	Space	Effective gate complexity
classical	zero temp mean-field with occ-RI-K/ACE [11, 12]	$N \le \Theta(\eta^3)$	$\widetilde{\mathcal{O}}(N\eta)$	$N^{4/3}\eta^{7/3}t(Nt/\epsilon)^{o(1)}$
classical	zero temp mean-field with occ-RI-K/ACE [11, 12]	$N \ge \Theta(\eta^3)$	$\widetilde{\mathcal{O}}(N\eta)$	$N^{5/3}\eta^{4/3}t(Nt/\epsilon)^{o(1)}$
quantum	second-quantized Trotter grid algorithm [1]	$N \le \Theta(\eta^2)$	$\mathcal{O}(N\log N)$	$N^{4/3}\eta^{1/3}t(Nt/\epsilon)^{o(1)}$
quantum	first-quantized Trotter grid algorithm here	$\Theta(\eta^2) \le N \le \Theta(\eta^3)$	$\mathcal{O}(\eta\log N)$	$N^{1/3}\eta^{7/3}t(Nt/\epsilon)^{o(1)}$
quantum	first-quantized Trotter grid algorithm here	$\Theta(\eta^3) \le N < \Theta(\eta^4)$	$\mathcal{O}(\eta\log N)$	$N^{2/3}\eta^{4/3}t(Nt/\epsilon)^{o(1)}$
quantum	qubitization algorithms from [5] or [6]	$N = \Theta(\eta^4)$	$\mathcal{O}(\eta\log N)$	$\widetilde{\mathcal{O}}(N^{2/3}\eta^{4/3}t)$
quantum	interaction picture algorithms from [5] or [6]	$N > \Theta(\eta^4)$	$\mathcal{O}(\eta\log N)$	$\widetilde{\mathcal{O}}(N^{1/3}\eta^{8/3}t)$

Supplementary Table 1: Best known gate complexities of exact quantum algorithms and classical mean-field algorithms for sampling the output of time-evolution, by ratio of basis size to particle number. Here we use the asymptotic $\Theta(\cdot)$ notation, which implies the union of both an asymptotic upper-bound and an asymptotic lower-bound on the scaling. N is number of basis functions, η is number of particles, ϵ is target precision, and t is duration of evolution. "Effective gate complexity" is the leading order scaling in the stated regime. All quantum algorithms discussed here require either a plane wave or grid basis. For those basis sets, the large space overhead of second quantization likely makes second-quantized approaches infeasible in practice. When $N = \eta^4$, the quantum algorithms with the best asymptotic scaling are the plane wave or grid basis qubitization algorithms from [5] or [6], respectively, as opposed to the interaction picture algorithms of those same works. This is due to lower polylogarithmic factors in the scaling that are suppressed by the $\widetilde{\mathcal{O}}(\cdot)$ notation.

classical random access memory (RAM) model. By contrast, the standard way of computing the Coulomb potential (involving summing up all $\binom{\eta}{2}$ pairs of electrons) scales as $\widetilde{\mathcal{O}}(\eta^2)$. Thus, if one can figure out how to extend these better scaling methods to first quantization with $\widetilde{\mathcal{O}}(\eta)$ operations in the reversible circuit model (the cost model of relevance for this subroutine if executed on a quantum computer), the quantum algorithm would scale as

$$\left(N^{1/3}\eta^{4/3}t + N^{2/3}\eta^{1/3}t\right)\left(\frac{Nt}{\epsilon}\right)^{o(1)} . \tag{33}$$

We note that it is straightforward to adapt these algorithms to second quantization with $\widetilde{\mathcal{O}}(N)$ gate complexity [1, 16]. However, translating such algorithms to first quantization with $\widetilde{\mathcal{O}}(N)$ gate complexity in the quantum circuit model is highly non-trivial. This is due to nuances of how adaptive tree-like data structures are constructed and used in these algorithms, and it is why the work of [8] decided to invoke the impractical assumption of QRAM in order to leverage the fast multipole algorithms. Note further that some of these algorithms such as the original fast multipole [13] and particle-mesh Ewald [15] make further assumptions on the state. In particular, if space is partitioned into $\mathcal{O}(\eta)$ boxes, then these methods require that no more than k electrons are present in any box, in any configuration on which the wavefunction has support. Since electrons tend to repel one another this is often a good assumption at low energies, but it is not true for general states. It seems possible to implement a first-quantized algorithm with $\widetilde{\mathcal{O}}(\eta\, k)$ space complexity and $\widetilde{\mathcal{O}}(\eta\,\mathrm{poly}(k))$ gate complexity by keeping k electron registers for each of these $\mathcal{O}(\eta)$ boxes of space. But there also exist versions of these algorithms, e.g. described in [17], which use RAM and an adaptive tree structure to give $\widetilde{\mathcal{O}}(\eta)$ complexity without any assumptions on the state. Such approaches appear quite challenging to port to the quantum circuit model with the same complexity. However, if possible, the first-quantized fast multipole-based Trotter would scale better than all other known approaches as long as $N < \eta^7$. When $N > \eta^7$, the first-quantized interaction picture algorithm has better scaling.

Supplementary Note 6 Efficient reduced density matrix estimation using classical shadows in first quantization

6.1 Problem statement

We consider a system of η identical fermions occupying $N \gg \eta$ orbitals. In first-quantization, we represent the state of such a system as a wavefunction on η registers of $n = \lceil \log(N) \rceil$ qubits. We demand that this wavefunction is antisymmetric under the exchange of any two registers in order for it to represent a valid physical state.

Most physically interesting observables of such a system are captured by the few-body marginals, the reduced density matrices. In this section, we concern ourselves with efficiently estimating elements of the k-body reduced density matrix (k-RDM) of the first-quantized state $|\psi\rangle$ defined on η identical fermion particles,

$$ {}^{k}D^{j_1,\ldots,j_k}_{i_1,\ldots,i_k} = \frac{\eta!}{(\eta-k)!} \operatorname{tr}\left[|\psi\rangle\langle\psi| \prod_{\ell=1}^{k} |i_\ell\rangle\langle j_\ell|_\ell \right], \tag{34} $$

where $|i\rangle\langle j|_\ell$ indicates the tensor product of $|i\rangle\langle j|$ on the ℓth register with the identity on the other $\eta - 1$ registers. We can write an equivalent definition (equivalent due to the antisymmetry of the wavefunction),

$$ {}^{k}D^{j_1,\ldots,j_k}_{i_1,\ldots,i_k} = \sum_{\boldsymbol{x} \in S_k^\eta} \operatorname{tr}\left[|\psi\rangle\langle\psi| \prod_{\ell=1}^{k} |i_\ell\rangle\langle j_\ell|_{x_\ell} \right], \tag{35} $$

where S_k^η is the set composed of all possible sequences of length k generating by drawing without replacement from $[\eta] := \{1, \ldots, \eta\}$.

Our goal is to use measurements of the state $|\psi\rangle$ to obtain a classical description of the state with enough information to approximate all N^{2k} elements of the k-RDM. We would like all of these estimates to accurate up to some additive error ϵ with probability at least $1 - \delta$. Ideally,

our protocol will be efficient not only in terms of the number of measurements, but also in terms of the (gate) complexity of implementing each measurement and the classical complexity of the required post-processing.

We will accomplish our goal by applying the classical shadows formalism of Ref. [18]. We propose and analyze a protocol that requires at most

$$m = 64e^3 \log\left(N/\delta\right) k \left(2k + 2e\right)^k \eta^k \epsilon^{-2} \tag{36}$$

measurements to estimate the k-RDM. Performing these measurements requires acting on each of the particle registers with a randomly sampled Clifford circuit and performing a measurement in the computational basis. These circuits can be implemented using $\mathcal{O}(\eta n^2)$ one- and two-qubit Clifford gates on a linearly connected array of qubits in depth $\mathcal{O}(n)$. Each element of the k-RDM requires performing a number of classical operations that scales as

$$m' = \mathcal{O}\left(\left(n^4 + \log\left(1/\delta\right)\right) \eta^{2k} 2^k \epsilon^{-2}\right). \tag{37}$$

Since the introduction of classical shadows in Ref. [18], several works have developed classical shadow protocols for estimating the expectation values of fermionic observables in second quantization [19, 20, 21, 22]. In particular, Ref. [22] recently proposed a technique that, like our approach, requires a number of samples that scales polylogarithmically in N to estimate all N^{2k} elements of the fermionic k-RDM. Ref. [22]'s approach and ours both make use of random unitaries drawn from efficiently simulable families of quantum circuits (fermionic Gaussian unitaries and Clifford unitaries respectively). A key difference between their work and ours is that our Clifford unitaries act on $n \sim \log(N)$ qubits, whereas the fermionic Gaussian unitaries of Ref. [22] act on N qubits. Thus, the approach of Ref. [22] does not seem like it would be feasible for the first-quantized representations that we focus on simulating in this work, since that would likely necessitate using millions of logical qubits.

Partly because of this difference, the measurement strategy we develop in this work has three key advantages over a translation of Ref. [22]'s techniques to the first-quantized setting. First of all, Ref. [22] requires the implementation of single particle basis changes that are specified by Haar random $n \times n$ unitaries. Performing these operations in first quantization would require a number of operations that scales exponentially with n (naively, $\mathcal{O}(N^2\eta)$ arbitrary rotations), whereas we can implement our random unitaries using only $\mathcal{O}(n^2\eta)$ Clifford gates. Secondly, and relatedly, the classical postprocessing required to estimate an element of the k-RDM using Ref. [22]'s shadow protocol scales exponentially with n, whereas ours scales polynomially. Thirdly, (and perhaps least significantly) we note that we are able to guarantee that the error on all estimated observables is less than ϵ with high probability. The bounds of Ref. [22] only promise that the average error across all elements of the k-RDM is bounded.

6.2 The measurement protocol

The classical shadows formalism of Huang et al. works by choosing an ensemble of random unitaries $\mathcal{U}$ on n qubits and defining a measurement channel that acts on a density matrix σ such that

$$\mathcal{M}(\sigma) := \mathbb{E}_{U \sim \mathcal{U}} \sum_{b \in \{0,1\}^n} U^\dagger |b\rangle\langle b| U \langle b|U\sigma U^\dagger|b\rangle . \tag{38}$$

For specific choices of $\mathcal{U}$, the channel $\mathcal{M}$ is analytically invertible. Operationally, we obtain the classical shadow of σ by repeatedly sampling a unitary U from $\mathcal{U}$, applying the sampled U to a copy of σ, and measuring in the computational basis (obtaining the bitstring b). If we collect m such samples, then we call the (potentially unphysical) state

$$\hat{\sigma} := \frac{1}{m} \sum_{i=1}^{m} \mathcal{M}^{-1} \left(U_i^\dagger |b_i\rangle\langle b_i| U_i \right) \tag{39}$$

a classical shadow of σ. For an arbitrary observable O, we can define an estimator $\hat{o}$ of the quantity $\mathrm{tr}\left[O\rho\right]$ using the classical shadow of ρ,

$$\hat{o} := \mathrm{tr}\left[O\hat{\rho}\right] . \tag{40}$$

In expectation, we have that

$$\langle\hat{\sigma}\rangle = \mathbb{E}_{U\sim\mathcal{U}} \sum_{b\in\{0,1\}^n} \mathcal{M}^{-1}\left(U^\dagger |b\rangle\langle b| U\right) \langle b|U\sigma U^\dagger|b\rangle = \mathcal{M}^{-1}\left(\mathcal{M}\left(\sigma\right)\right) = \sigma. \tag{41}$$

When we take $\mathcal{U}$ to be the uniform distribution over the Clifford group on n qubits, the classical shadows measurement channel and its inverse have particularly simple forms [18],[1]

$$\mathcal{M}(A) = \frac{1}{2^n+1}A + \frac{\mathrm{tr}\left[A\right]}{2^n+1}\mathbb{I}, \tag{42}$$

$$\mathcal{M}^{-1}(A) = (2^n+1)A - \mathrm{tr}\left[A\right]\mathbb{I}. \tag{43}$$

Here, and throughout our analysis of the measurement protocol, we use the symbol $\mathbb{I}$ to denote the identity operator on a Hilbert space whose dimension is appropriate for the context.

In this work, we propose and analyze the impact of using an ensemble $\mathcal{U}$ that consists of a tensor product of η copies of the uniform distribution over n qubit Clifford circuits,

$$\mathcal{U} = \bigotimes_{j=1}^{\eta} \mathrm{Cl}(2^n). \tag{44}$$

That is to say, we perform our measurements by independently sampling η n-qubit Clifford unitaries, applying one to each particle register, and measuring in the computational basis. We can consider the action of the corresponding classical shadow measurement channel and its inverse on an operator $X_1 \otimes \cdots \otimes X_\eta$ that factorizes across the η registers. The channel is defined on the whole Hilbert space by linear extension. For the classical shadow measurement channel, we have

$$\mathcal{M}(X_1\otimes\cdots\otimes X_\eta) = \bigotimes_{j=1}^{\eta}\left(\mathbb{E}_{U_j\sim\mathrm{Cl}(2^n)} \sum_{b_j\in\{0,1\}^n} U_j^\dagger |b_j\rangle\langle b_j| U_j \langle b_j|U_j X_j U_j^\dagger|b_j\rangle\right) \tag{45}$$

$$= \bigotimes_{j=1}^{\eta}\left(\frac{X_j + \mathrm{tr}\left[X_j\right]\mathbb{I}}{2^n+1}\right). \tag{46}$$

[1]Actually, a substantial constant factor savings in the number of gates can be obtained by using the canonical form of Ref. [23] and simply dropping the permutation at the end of the circuit. See, e.g., Ref. [24].

The inverse, similarly, is given by

$$\mathcal{M}^{-1}(X_1 \otimes \cdots \otimes X_\eta) = \bigotimes_{j=1}^{\eta} \left(\left(2^n + 1\right) X_j - \operatorname{tr}\left[X_j\right] \mathbb{I} \right). \tag{47}$$

Due to the antisymmetry of the wavefunction, we have the freedom to choose between a number of different observables when estimating the elements of the k-RDM. Consider an arbitrary operator O, and the operator $POP^\dagger$, where P is an operator that permutes the particle registers. The expectation values of O and $POP^\dagger$ with respect to a first-quantized wavefunction are the same (to see this, observe that any sign picked up by acting $P^\dagger$ on the ket is cancelled out by a corresponding sign obtained from acting P on the bra). We can use this degree of freedom to minimize the variance of our measurement protocol. Using the observable from Eq. (34) to construct a classical shadow estimator of a k-RDM element would lead to an unnecessarily large variance, essentially because the observable doesn't take advantage of all of the information present in the state. In contrast, Eq. (35) defines the k-RDM element in terms of a sum over many different permutations of the registers. We conjecture that a measurement protocol based on the observable in Eq. (35) would perform well, but the analysis could be tedious due to the many different cases that would arise.

Rather than using the observables implied by either Eq. (34) or Eq. (35) in our classical shadow measurement procedure, we instead choose to estimate the k-RDM elements using an observable that involves a sum over a simpler set of permutations. Essentially, we break the η registers up into k groups of size η/k and measure the k-RDM element using registers from each group. For ease of notation, let us assume that η is divisible by k.[2] Formally, we can define a set of sequences

$$R_k = \{1, \ldots, \eta/k\} \times \{\eta/k + 1, \ldots, 2\eta/k\} \times \cdots \times \{(k-1)\,\eta/k + 1, \ldots, \eta\}. \tag{48}$$

[2] In the event that η is not exactly divisible by k, one could modify the protocol to either use groups of slightly different sizes or to only perform the measurements using $\eta' = k\lfloor \eta/k \rfloor$ registers.

Due to the antisymmetry of the wavefunction, we have that

$$
{}^{k}D^{j_1,\ldots,j_k}_{i_1,\ldots,i_k} = \frac{k^k\,(\eta!)}{\eta^k\,(\eta-k)!}\sum_{\boldsymbol{x}\in R_k}\mathrm{tr}\left[|\psi\rangle\langle\psi|\prod_{\ell=1}^{k}|i_\ell\rangle\langle j_\ell|_{x_\ell}\right]. \tag{49}
$$

We define an estimator $\hat{d}$ for the k-RDM element ${}^{k}D^{j_1,\ldots,j_k}_{i_1,\ldots,i_k}$ using the classical shadow $\hat{\rho}$ of $|\psi\rangle$,

$$
\hat{d} = \frac{k^k\,(\eta!)}{\eta^k\,(\eta-k)!}\sum_{\boldsymbol{x}\in R_k}\mathrm{tr}\left[\hat{\rho}\prod_{\ell=1}^{k}|i_\ell\rangle\langle j_\ell|_{x_\ell}\right]. \tag{50}
$$

In Supplementary Note 6.4, we prove that the single-shot variance of this estimator is bounded by

$$
\mathrm{Var}(\hat{d}) \le e^3\eta^k\,(2k+2e)^k\,. \tag{51}
$$

In order to guarantee that our estimates are close to the true value of the k-RDM elements with high probability, we need to proceed along the same lines as Ref. [18] and construct a median-of-means estimator to obtain the desired rigorous guarantees [25]. To be precise, using Proposition 12 from Ref. [25], we can consider an estimator that divides the m total classical shadow samples into K groups of size b, and takes the median of the sample mean obtained by averaging the estimates within each group. The probability that this median of means estimator has an error larger than $2\sqrt{\mathrm{Var}(\hat{d})/b}$ is at most $e^{-K/8}$. To bound the error in our estimate by ϵ with a success probability of at least $1-\delta$, this implies that we need

$$
b = 4\,\mathrm{Var}(\hat{d})/\epsilon^2, \tag{52}
$$

$$
K = 8\log\left(1/\delta\right). \tag{53}
$$

The overall number of measurements claimed in Eq. (36) follows directly from applying a union bound over the failure probabilities for estimating all N^{2k} k-RDM elements.

The measurement protocol can be summarized as follows. We take a classical shadow of $|\psi\rangle$ with the $\mathcal{U}$ defined in Eq. (44) using a number of samples m chosen according to Eq. (36). For

each sample, we evaluate the expectation values of the $(\eta/k)^k$ different terms in the sum over R_k (see Eq. (50)) using generalizations of Gottesman-Knill theorem that account for the phase of the quantities involved [26, 27, 28]. Breaking the samples into K groups of size b, averaging within the groups, and then taking the median of these means then yields the final estimate. The classical post-processing costs quoted in Eq. (37) come from counting the number of n-qubit sized Clifford circuits that need to be simulated classically to carry out this procedure.

6.3 Notation and preliminaries

Before we proceed to bound the variance of the estimator $\hat{d}$ for an arbitrary k-RDM element, it is helpful to recall a few useful expressions and prove some identities that we will use later.

We will make use of a formula for the two-fold twirl over the Clifford group and partial trace obtained from Ref. [18],

$$\mathbb{E}_{U\sim\mathrm{Cl}(2^n)} U^\dagger |x\rangle\langle x| U \langle x|UAU^\dagger|x\rangle = \frac{A + \mathrm{tr}(A)\mathbb{I}}{2^n(2^n+1)}. \tag{54}$$

For the three-fold twirl and partial trace, we find it convenient to use the identity

$$\mathbb{E}_{U\sim\mathrm{Cl}(2^n)} U^\dagger |x\rangle\langle x| U \langle x|UBU^\dagger|x\rangle \ \langle x|UCU^\dagger|x\rangle = \frac{1}{2^n(2^n+1)(2^n+2)} \left(\mathbb{I}\left(\mathrm{tr}\left[BC\right] + \mathrm{tr}\left[B\right]\mathrm{tr}\left[C\right]\right) + B\,\mathrm{tr}\left[C\right] + C\,\mathrm{tr}\left[B\right] + BC + CB\right). \tag{55}$$

This equation is different from the corresponding one considered in previous work (Eq. (S36) of Ref. [18]), in that it allows for B and C to have non-zero trace. It can be obtained directly from the analysis of Ref. [29].[3]

Another small departure we make from some prior work is that we directly consider the variance of estimators for the expectation values of non-Hermitian observables. For a classical shadow $\hat{\rho}$ of a state ρ and an estimator $\hat{o} = \mathrm{tr}\left[\hat{\rho}O\right]$ of the expectation value of a (not necessarily Hermitian)

[3] Note that while the proof of Lemma 7 in Ref. [29] is technically for Hermitian matrices, the same proof holds exactly in the non-Hermitian case.

operator O, we have

$$\mathrm{Var}(\hat{o}) = \mathrm{tr}\left[\rho \sum_b \mathbb{E}_{U\sim\mathcal{U}} U^\dagger |b\rangle\langle b| U \langle b|U\mathcal{M}^{-1}(O)U^\dagger|b\rangle \langle b|U\mathcal{M}^{-1}(O^\dagger)U^\dagger|b\rangle\right] - |\mathrm{tr}\left[O\rho\right]|^2 \tag{56}$$

$$\leq \mathrm{tr}\left[\rho \sum_b \mathbb{E}_{U\sim\mathcal{U}} U^\dagger |b\rangle\langle b| U \langle b|U\mathcal{M}^{-1}(O)U^\dagger|b\rangle \langle b|U\mathcal{M}^{-1}(O^\dagger)U^\dagger|b\rangle\right]. \tag{57}$$

This expression can be arrived at from the definition of the variance of a complex-valued random variable applied to the classical shadow formalism. We refer the reader to Ref. [20] for a thorough discussion.

In the course of calculating the variance for the higher-order RDMs, we will find that we repeatedly need to simplify certain expressions. Before describing those expressions and showing how they may be simplified, let us define some notation used for convenience throughout the rest of our analysis:

$$P_x = |x\rangle\langle x|, \tag{58}$$

$$P_{xy} = |x\rangle\langle y|, \tag{59}$$

$$\mathbb{E}_U = \mathbb{E}_{U\sim \mathrm{Cl}(2^n)}, \tag{60}$$

$$\sum_b = \sum_{b\in\{0,1\}^n}. \tag{61}$$

One class of expressions that we will need to simplify are of the form

$$A = \mathbb{E}_U \sum_b U^\dagger P_b U \langle b|U\mathcal{M}^{-1}(P_{ij})U^\dagger|b\rangle. \tag{62}$$

We can use Eq. (43) and Eq. (54) to simplify Eq. (62),

$$A = \mathbb{E}_U \sum_b U^\dagger P_b U \langle b|U\mathcal{M}^{-1}(P_{ij})U^\dagger|b\rangle \tag{63}$$

$$= \mathbb{E}_U \sum_b U^\dagger P_b U \langle b|U\left(\left(2^n+1\right)P_{ij} - \delta_{i,j}\mathbb{I}\right)U^\dagger|b\rangle \tag{64}$$

$$= P_{ij}. \tag{65}$$

Another kind of expression that we will need to simplify is of the form

$$A = \mathbb{E}_U \sum_b U^\dagger P_b U \left\langle b|U\mathcal{M}^{-1}(P_{ij})U^\dagger|b\right\rangle \left\langle b|U\mathcal{M}^{-1}(P_{kl})U^\dagger|b\right\rangle . \tag{66}$$

Let us consider the first case, and simplify A as defined below,

$$A = \mathbb{E}_U \sum_b U^\dagger P_b U \left\langle b|U\mathcal{M}^{-1}(P_i)U^\dagger|b\right\rangle \left\langle b|U\mathcal{M}^{-1}(P_i)U^\dagger|b\right\rangle . \tag{67}$$

We have

$$\mathcal{M}^{-1}\left(P_i\right) = \left(2^n+1\right)P_i - \mathbb{I} \tag{68}$$

by an application of Eq. (43). Now we can apply Eq. (55) with $B = C = \left(2^n+1\right)P_i - \mathbb{I}$.

Let us simplify the pieces of Eq. (55) separately before combining them. We have

$$BC = CB = \left(\left(2^n+1\right)P_i - \mathbb{I}\right)^2 \tag{69}$$

$$= \left(2^n+1\right)\left(2^n-1\right)P_i + \mathbb{I}, \tag{70}$$

$$\operatorname{tr}\left[BC\right] = \operatorname{tr}\left[CB\right] = 2^n(2^n+1) - 1, \tag{71}$$

$$\operatorname{tr}\left[B\right] = \operatorname{tr}\left[C\right] = 1. \tag{72}$$

As a result,

$$\mathbb{I}\left(\operatorname{tr}\left[BC\right] + \operatorname{tr}\left[B\right]\operatorname{tr}\left[C\right]\right) + B\operatorname{tr}\left[C\right] + C\operatorname{tr}\left[B\right] + BC + CB \tag{73}$$

$$= 2^n\left(2^n+1\right)\mathbb{I} + 2\left(2^n+1\right)P_i - 2\mathbb{I} + 2\left(2^n+1\right)\left(2^n-1\right)P_i + 2\mathbb{I} \tag{74}$$

$$= 2^n\left(2^n+1\right)\left(\mathbb{I} + 2P_i\right). \tag{75}$$

Putting everything together, we have

$$A = \mathbb{E}_U \sum_b U^\dagger P_b U \left\langle b|U\mathcal{M}^{-1}(P_i)U^\dagger|b\right\rangle \left\langle b|U\mathcal{M}^{-1}(P_i)U^\dagger|b\right\rangle \tag{76}$$

$$= \frac{2^n}{2^n+2}\left(\mathbb{I} + 2P_i\right). \tag{77}$$

Now we consider simplifying the expression

$$A = \mathbb{E}_U \sum_b U^\dagger P_b U \langle b|U\mathcal{M}^{-1}(P_{ij})U^\dagger|b\rangle \; \langle b|U\mathcal{M}^{-1}(P_{ji})U^\dagger|b\rangle \,. \tag{78}$$

In this case, we can again use Eq. (55) with

$$B = \mathcal{M}^{-1}(P_{ij}) = (2^n+1)\, P_{ij}, \tag{79}$$

$$C = \mathcal{M}^{-1}(P_{ji}) = (2^n+1)\, P_{ji}. \tag{80}$$

Working out some of the pieces, we have

$$BC = (2^n+1)^2\, P_i, \tag{81}$$

$$CB = (2^n+1)^2\, P_j, \tag{82}$$

$$\operatorname{tr}[BC] = (2^n+1)^2\,, \tag{83}$$

$$\operatorname{tr}[B] = \operatorname{tr}[C] = 0. \tag{84}$$

Therefore,

$$\mathbb{I}\left(\operatorname{tr}[BC] + \operatorname{tr}[B]\operatorname{tr}[C]\right) + B\operatorname{tr}[C] + C\operatorname{tr}[B] + BC + CB \tag{85}$$

$$= (2^n+1)^2\left(\mathbb{I} + P_i + P_j\right). \tag{86}$$

Finally, we have

$$A = \mathbb{E}_U \sum_b U^\dagger P_b U \langle b|U\mathcal{M}^{-1}(P_{ij})U^\dagger|b\rangle \; \langle b|U\mathcal{M}^{-1}(P_{ji})U^\dagger|b\rangle \tag{87}$$

$$= \frac{2^n+1}{2^n+2}\left(\mathbb{I} + P_i + P_j\right). \tag{88}$$

6.4 Variance of the k-RDM with a restricted sum

Now we are ready to turn to the task of bounding the variance $\hat{d}$ as defined in Eq. (50). For now, we neglect the coefficient in order to simplify the presentation. Let

$$O = \sum_{\boldsymbol{x} \in R_k} O_{\boldsymbol{x}}, \tag{89}$$

$$O_{\boldsymbol{x}} = \prod_{\ell=1}^{k} |i_\ell\rangle\langle j_\ell|_{x_\ell}. \tag{90}$$

The variance of the classical shadow estimator $\hat{o}$ of $\langle O \rangle$ is bounded by

$$\mathrm{Var}(\hat{o}) \leq \sum_{\boldsymbol{x} \in R_k} \sum_{\boldsymbol{y} \in R_k} \mathrm{tr}\left[|\psi\rangle\langle\psi| A_{\boldsymbol{xy}}\right], \tag{91}$$

$$A_{\boldsymbol{xy}} = \sum_b \mathbb{E}_{U \sim \mathcal{U}} U^\dagger |b\rangle\langle b| U \langle b|U\mathcal{M}^{-1}(O_{\boldsymbol{x}})U^\dagger|b\rangle \langle b|U\mathcal{M}^{-1}(O_{\boldsymbol{y}}^\dagger)U^\dagger|b\rangle. \tag{92}$$

Because the inverse channel, the random unitaries, and the $O_{\boldsymbol{x}}$ all factorize across the registers, we can rewrite $A_{\boldsymbol{xy}}$ as a tensor product,

$$A_{\boldsymbol{xy}} = \bigotimes_{z=1}^{\eta} A_{\boldsymbol{xy}}^z, \tag{93}$$

where $A_{\boldsymbol{xy}}^z$ takes one of three forms depending on whether neither, one of, or both of $O_{\boldsymbol{x}}$ and $O_{\boldsymbol{y}}$ act non-trivially on the zth register. If $z \notin \boldsymbol{x}$ and $z \notin \boldsymbol{y}$, then

$$A_{\boldsymbol{xy}}^z = \mathbb{I}. \tag{94}$$

If exactly one of $z \in \boldsymbol{x}$ or $z \in \boldsymbol{y}$ is true, then we can use Eq. (65) to simplify our expression for $A_{\boldsymbol{xy}}^z$. The cases are symmetric between $z \in \boldsymbol{x}$ and $z \in \boldsymbol{y}$, so we can treat only the first case without loss of generality. Let ℓ denote the index of z in $\boldsymbol{x}$ (i.e., $x_\ell = z$). We have

$$A_{\boldsymbol{xy}}^z = \sum_b \mathbb{E}_{U \sim \mathcal{U}} U^\dagger |b\rangle\langle b| U \langle b|U\mathcal{M}^{-1}(|i_\ell\rangle\langle j_\ell|)U^\dagger|b\rangle \tag{95}$$

$$= |i_\ell\rangle\langle j_\ell|_z. \tag{96}$$

If $z \in \boldsymbol{y}$ we instead have $A^z_{\boldsymbol{x}\boldsymbol{y}} = |j_\ell\rangle\langle i_\ell|_z$.

The third case we must consider is where $z \in \boldsymbol{x}$ and $z \in \boldsymbol{y}$. Let ℓ denote the index of z in $\boldsymbol{x}$ and $\boldsymbol{y}$ (they must be the same because of the way we construct $\boldsymbol{x}$ and $\boldsymbol{y}$). In this case,

$$A^z_{\boldsymbol{x}\boldsymbol{y}} = \sum_b \mathbb{E}_{U\sim\mathcal{U}} U^\dagger |b\rangle\langle b| U \langle b|U\mathcal{M}^{-1}(|i_\ell\rangle\langle j_\ell|)U^\dagger|b\rangle\, U \langle b|U\mathcal{M}^{-1}(|j_\ell\rangle\langle i_\ell|)U^\dagger|b\rangle\,. \tag{97}$$

If $i_\ell = j_\ell$ we can simplify this expression using Eq. (77), otherwise we can use Eq. (88). The combination of these two formulas lets us write

$$\begin{aligned} A^z_{\boldsymbol{x}\boldsymbol{y}} &= \sum_b \mathbb{E}_{U\sim\mathcal{U}} U^\dagger |b\rangle\langle b| U \langle b|U\mathcal{M}^{-1}(|i_\ell\rangle\langle j_\ell|)U^\dagger|b\rangle\, U \langle b|U\mathcal{M}^{-1}(|j_\ell\rangle\langle i_\ell|)U^\dagger|b\rangle & (98)\\ &= \frac{2^n + 1 - \delta_{i_\ell,j_\ell}}{2^n+2}\left(\mathbb{I} + |i_\ell\rangle\langle i_\ell| + |j_\ell\rangle\langle j_\ell|\right). & (99)\end{aligned}$$

Now we will use the antisymmetry of $|\psi\rangle$ to bound the quantity $|\mathrm{tr}\left[|\psi\rangle\langle\psi|\, A_{\boldsymbol{x}\boldsymbol{y}}\right]|$. Let

$$a = |\boldsymbol{x} \cap \boldsymbol{y}|, \qquad b = 2k - 2a. \tag{100}$$

The operator $A_{\boldsymbol{x}\boldsymbol{y}}$ acts non-trivially on $a + b$ registers. On a registers, it acts with an operator of the form given in Eq. (99). On the other b registers, it acts as $|c\rangle\langle d|$ for some c, d (that can vary per register). Due to the antisymmetry of $|\psi\rangle$, we can freely permute the registers without affecting the expectation value.

We can therefore rewrite the expectation value of interest as

$$\begin{aligned} |\mathrm{tr}\left[|\psi\rangle\langle\psi|\, A_{\boldsymbol{x}\boldsymbol{y}}\right]| &= \left| \langle\psi| \left(\bigotimes_{\ell=1}^{a} \frac{2^n + 1 - \delta_{c_\ell,d_\ell}}{2^n+2}\left(\mathbb{I} + |c_\ell\rangle\langle c_\ell| + |d_\ell\rangle\langle d_\ell|\right) \bigotimes_{\ell=a+1}^{a+b} |c_\ell\rangle\langle d_\ell| \bigotimes_{\ell=a+b+1}^{\eta} \mathbb{I} \right) |\psi\rangle \right| & (101)\\ &\leq \left| \langle\psi| \left(\bigotimes_{\ell=1}^{a} \left(\mathbb{I} + |c_\ell\rangle\langle c_\ell| + |d_\ell\rangle\langle d_\ell|\right) \bigotimes_{\ell=a+1}^{a+b} |c_\ell\rangle\langle d_\ell| \bigotimes_{\ell=a+b+1}^{\eta} \mathbb{I} \right) |\psi\rangle \right|. & (102)\end{aligned}$$

6.4.1 Removing the off-diagonal terms

We can simplify the bound in Eq. (102) by replacing the off-diagonal matrix elements with projectors. To do so, we will need the following lemma.

Lemma 1. *Let $|\psi\rangle$ be an arbitrary normalized pure quantum state on n qubits. Let O be an arbitrary positive semidefinite operator on a qubits, and let $|\alpha\rangle$ and $|\beta\rangle$ be arbitrary orthonormal quantum states on $n-a$ qubits. Then,*

$$|\langle\psi|(O\otimes|\alpha\rangle\langle\beta|)|\psi\rangle| \leq |\langle\psi|(O\otimes|\phi\rangle\langle\phi|)|\psi\rangle| \tag{103}$$

for $|\phi\rangle = |\alpha\rangle$ or $|\phi\rangle = |\beta\rangle$.

Proof. To begin the proof, expand $|\psi\rangle$ as

$$|\psi\rangle = \sum_{ij} c_{ij}\,|i\rangle\,|j\rangle\,, \tag{104}$$

where the states $\{|i\rangle\}$ form an eigenbasis for O and the states $\{|j\rangle\}$ are an orthonormal basis such that $|\alpha\rangle\,, |\beta\rangle \in \{|j\rangle\}$. Then

$$|\langle\psi|(O\otimes|\alpha\rangle\langle\beta|)|\psi\rangle| = \left|\sum_i c_{i\alpha}^* c_{i\beta} O_{ii}\right| \tag{105}$$

$$= \sum_i k_{i\alpha}^* k_{i\beta}, \tag{106}$$

where O_{ii} denotes the eigenvalue of O corresponding to the eigenvector $|i\rangle$ and k_{ij} is defined implicitly as $k_{ij} = c_{ij}\sqrt{O_{ii}}$. We can consider the quantity in Eq. (106) as the inner product of two vectors $\vec{k}_\alpha$ and $\vec{k}_\beta$. The Cauchy-Schwarz inequality tells us that

$$\left|\sum_i k_{i\alpha}^* k_{i\beta}\right| \leq \sqrt{\left(\sum_i k_{i\alpha}^* k_{i\alpha}\right)\left(\sum_i k_{i\beta}^* k_{i\beta}\right)}. \tag{107}$$

We can choose $\gamma \in \{\alpha, \beta\}$ such that

$$\left|\sum_i k_{i\gamma}^* k_{i\gamma}\right| \geq \left|\sum_i k_{i\alpha}^* k_{i\alpha}\right| \text{ and}$$
$$\left|\sum_i k_{i\gamma}^* k_{i\gamma}\right| \geq \left|\sum_i k_{i\beta}^* k_{i\beta}\right|. \tag{108}$$

Therefore, we have that

$$|\langle\psi|(O \otimes |\alpha\rangle\langle\beta|)|\psi\rangle| \leq \left|\sum_i k_{i\gamma}^* k_{i\gamma}\right| \tag{109}$$
$$= \left|\sum_i c_{i\gamma}^* c_{i\gamma} O_{ii}\right| \tag{110}$$
$$= \langle\psi|(O \otimes |\gamma\rangle\langle\gamma|)|\psi\rangle \tag{111}$$

for either $|\gamma\rangle = |\alpha\rangle$ or $|\gamma\rangle = |\beta\rangle$ We can remove the absolute value bars in the final line because $O \otimes |\gamma\rangle\langle\gamma|$ is a positive semidefinite operator. □

Now we can return to our bound from Eq. (102),

$$|\mathrm{tr}\left[|\psi\rangle\langle\psi| A_{\boldsymbol{xy}}\right]| \leq \left|\langle\psi|\left(\bigotimes_{\ell=1}^{a}(\mathbb{I} + |c_\ell\rangle\langle c_\ell| + |d_\ell\rangle\langle d_\ell|)\bigotimes_{\ell=a+1}^{a+b}|c_\ell\rangle\langle d_\ell|\bigotimes_{\ell=a+b+1}^{\eta}\mathbb{I}\right)|\psi\rangle\right|. \tag{112}$$

By rearranging the registers, we can apply Lemma 1. Taking $|\alpha\rangle$ to be $\bigotimes_{\ell=a+1}^{a+b}|c_\ell\rangle$ and $\langle\beta|$ to be $\bigotimes_{\ell=a+1}^{a+b}\langle d_\ell|$, we can show that either

$$|\mathrm{tr}\left[|\psi\rangle\langle\psi| A_{\boldsymbol{xy}}\right]| \leq \langle\psi|\left(\bigotimes_{\ell=1}^{a}(\mathbb{I} + |c_\ell\rangle\langle c_\ell| + |d_\ell\rangle\langle d_\ell|)\bigotimes_{\ell=a+1}^{a+b}|c_\ell\rangle\langle c_\ell|\bigotimes_{\ell=a+b+1}^{\eta}\mathbb{I}\right)|\psi\rangle \tag{113}$$

holds, or an equivalent expression with $|d_\ell\rangle\langle d_\ell|$ instead of $|c_\ell\rangle\langle c_\ell|$ in the second set of registers. Both cases are identical, so we will proceed using the label g_ℓ for whichever choice is valid in each register.

We can also simplify the expression in the first registers. We claim that, for each register, we can replace the term $|c_\ell\rangle\langle c_\ell| + |d_\ell\rangle\langle d_\ell|$ with either $2|c_\ell\rangle\langle c_\ell|$ or $2|d_\ell\rangle\langle d_\ell|$ without making

the expectation value any smaller. This can be seen by proceeding register by register, using the linearity of the expectation value. Here again, the choice of c_ℓ or d_ℓ in each register is immaterial, so we use the label g_ℓ to denote whichever one is appropriate for each register. Making this simplification, we have that

$$|\mathrm{tr}\left[|\psi\rangle\langle\psi|\, A_{\boldsymbol{xy}}\right]| \leq \langle\psi|\left(\bigotimes_{\ell=1}^{a}(\mathbb{I}+2\,|g_\ell\rangle\langle g_\ell|)\bigotimes_{\ell=a+1}^{a+b}|g_\ell\rangle\langle g_\ell|\bigotimes_{\ell=a+b+1}^{\eta}\mathbb{I}\right)|\psi\rangle\,. \tag{114}$$

6.4.2 Taking advantage of antisymmetry

Now we will take advantage of the antisymmetry of $|\psi\rangle$ to bound the expectation values in Eq. (114). It is helpful to rewrite the expression in the first set of registers in a different form:

$$\bigotimes_{\ell=1}^{a}(\mathbb{I}+2\,|g_\ell\rangle\langle g_\ell|) = \sum_{w=0}^{a}2^w\sum_{S\subseteq[a]:|S|=w}\bigotimes_{\ell=1}^{a}W_\ell^S, \tag{115}$$

where $W_\ell^S = |g_\ell\rangle\langle g_\ell|$ if $\ell \in S$ and $W_\ell = \mathbb{I}$ otherwise. This then leads us to the bound

$$|\mathrm{tr}\left[|\psi\rangle\langle\psi|\, A_{\boldsymbol{xy}}\right]| \leq \sum_{w=0}^{a}2^w\sum_{S\subseteq[a]:|S|=w}\langle\psi|\left(\bigotimes_{\ell=1}^{a}W_\ell^S\bigotimes_{\ell=a+1}^{a+b}|g_\ell\rangle\langle g_\ell|\bigotimes_{\ell=a+b+1}^{\eta}\mathbb{I}\right)|\psi\rangle\,. \tag{116}$$

Now that we have obtained this bound, we will proceed to use the antisymmetry of $|\psi\rangle$ to show that

$$\langle\psi|\left(\bigotimes_{\ell=1}^{a}W_\ell^S,\bigotimes_{\ell=a+1}^{a+b}|g_\ell\rangle\langle g_\ell|\bigotimes_{\ell=a+b+1}^{\eta}\mathbb{I}\right)|\psi\rangle \leq \frac{1}{P(\eta,|S|+b)} = \frac{(\eta-|S|-b)!}{\eta!}. \tag{117}$$

To do so, let us prove the following lemma,

Lemma 2. *Let $|\psi\rangle$ be a normalized pure state on η registers of n qubits each. Furthermore, let $S\,|\psi\rangle = -\,|\psi\rangle$ for any operator S that swaps the states of two of the registers. Let $\{P_i\}_{i\in[k]}$ be a set of projectors onto orthonormal n qubit states. Then*

$$0 \leq \langle\psi|\left(\bigotimes_{i=1}^{k}P_i\bigotimes_{i=k+1}^{\eta}\mathbb{I}\right)|\psi\rangle \leq \frac{1}{P(\eta,k)} = \frac{(\eta-k)!}{\eta!}, \tag{118}$$

where $P(\eta,k)$ denotes the number of ways to choose a sequence of k items from a set of size η.

Proof. Let S_k denote the set of all sequences obtained by choosing k items from the set $[\eta]$. Note that two sequences with the same elements in different orders are treated as distinct elements of S_k. For a sequence $s \in S_k$ we define the operator A_s as the operator that acts on register s_i with the projector P_i for all $i \in [k]$ and acts on the other $\eta - k$ registers with the identity operation. Note that all of the operators A_s are defined using the same set of k projectors acting on (potentially) different registers.

We will prove the claim by showing that

$$\sum_{s \in S_k} \langle \psi | A_s | \psi \rangle \leq 1. \tag{119}$$

Clearly the operators $\{A_s\}_{s \in S_k}$ are all projectors onto different subspaces. In general, these projectors are not orthogonal (under the Hilbert-Schmidt inner product). Equivalently, we could say that the $+1$ eigenspaces of these operators are not orthogonal in general.

However, we can show that $|\psi\rangle$ has no support on states that are in the $+1$ eigenspace of more than one of these projectors. Consider A_x and A_y for $x \neq y$. There must be some register ℓ on which they act differently. If A_x and A_y both act on register ℓ with distinct projectors P_i and P_j then $A_x A_y$ = 0 and their eigenspaces have no overlap, so we are done. Assume that only one of A_x and A_y acts on register ℓ. Without loss of generality we consider the case where A_x acts on register ℓ with the projector P_i. Then, by definition, A_y acts on a different register ℓ' with P_i (since A_y acts with exactly the same projectors as A_x, just on a potentially different set of registers). Due to the antisymmetry of $|\psi\rangle$, we therefore have $\langle \psi | A_x A_y | \psi \rangle = 0$.

Therefore, we can assert that

$$\sum_{s \in S_k} \langle \psi | A_s | \psi \rangle \leq 1. \tag{120}$$

This could be seen in more detail by expanding $|\psi\rangle$ in the basis that diagonalizes all of the $\{A_s\}$ and applying the fact that if $A_x |\phi\rangle = 1$ then $A_y |\phi\rangle = 0$ for all $x \neq y$. The antisymmetry of $|\psi\rangle$

also implies that $\langle\psi|A_x|\psi\rangle = \langle\psi|A_y|\psi\rangle$ for all x, y. Therefore, we have that

$$|S_k| \langle\psi|A_s|\psi\rangle \leq 1 \tag{121}$$

for any A_s. The $\{A_s\}$ are all positive semidefinite, so we can bound the expectation value of the particular one from Eq. (118) below by zero and divide by $|S_k| = P(\eta, k)$ to yield

$$0 \leq \langle\psi| \left(\bigotimes_{i=1}^{k} P_i \bigotimes_{i=k+1}^{\eta} \mathbb{I} \right) |\psi\rangle \leq \frac{1}{P(\eta, k)} = \frac{(\eta - k)!}{\eta!}, \tag{122}$$

completing the proof. □

Eq. (117) follows directly from this lemma and the fact that we can freely permute the observables between registers without changing the expectation value. Now we can return to Eq. (116) and apply Eq. (117) to show that

$$|\mathrm{tr}\left[|\psi\rangle\langle\psi| A_{\boldsymbol{xy}}\right]| \leq \sum_{w=0}^{a} 2^w \sum_{S\subseteq[a]:|S|=w} \langle\psi| \left(\bigotimes_{\ell=1}^{a} W_\ell^S \bigotimes_{\ell=a+1}^{a+b} |g_\ell\rangle\langle g_\ell| \bigotimes_{\ell=a+b+1}^{\eta} \mathbb{I} \right) |\psi\rangle \tag{123}$$

$$\leq \sum_{w=0}^{a} 2^w \sum_{S\subseteq[a]:|S|=w} \frac{(\eta - w - b)!}{\eta!} \tag{124}$$

$$= \sum_{w=0}^{a} 2^w \sum_{S\subseteq[a]:|S|=w} \frac{(\eta - w)!}{\eta!} \frac{(\eta - w - b)!}{(\eta - w)!} \tag{125}$$

$$\leq \sum_{w=0}^{a} 2^w \sum_{S\subseteq[a]:|S|=w} \frac{(\eta - w)!}{\eta!} \frac{(\eta - a - b)!}{(\eta - a)!}, \tag{126}$$

with the last inequality following from the fact that $\eta - a \leq \eta - w$. Then we have that

$$|\mathrm{tr}\left[|\psi\rangle\langle\psi| A_{\boldsymbol{xy}}\right]| \leq \sum_{w=0}^{a} 2^w \sum_{S\subseteq[a]:|S|=w} \frac{(\eta-w)!}{\eta!}\frac{(\eta-a-b)!}{(\eta-a)!}, \tag{127}$$

$$= \frac{(\eta-a-b)!}{(\eta-a)!}\sum_{w=0}^{a} 2^w \sum_{S\subseteq[a]:|S|=w} \frac{(\eta-w)!}{\eta!} \tag{128}$$

$$= \frac{(\eta-a-b)!}{(\eta-a)!}\sum_{w=0}^{a} 2^w \binom{a}{w}\frac{(\eta-w)!}{\eta!} \tag{129}$$

$$\leq \frac{(\eta-a-b)!}{(\eta-a)!}\sum_{w=0}^{a} 2^w \binom{a}{w}\frac{(a-w)!}{a!} \tag{130}$$

$$= \frac{(\eta-a-b)!}{(\eta-a)!}\sum_{w=0}^{a} \frac{2^w}{w!} \tag{131}$$

$$\leq \frac{(\eta-a-b)!}{(\eta-a)!}\sum_{w=0}^{\infty} \frac{2^w}{w!} \tag{132}$$

$$= \frac{(\eta-a-b)!}{(\eta-a)!}e^2, \tag{133}$$

where the last step is obtained by the application of a well-known formula for the infinite sum of the sequence in Eq. (132).

6.4.3 Putting the pieces together

Having shown that

$$|\mathrm{tr}\left[|\psi\rangle\langle\psi| A_{\boldsymbol{xy}}\right]| \leq \frac{e^2(\eta-a-b)!}{(\eta-a)!}, \tag{134}$$

we are ready to return to the bound in Eq. (91), which we recall below:

$$\mathrm{Var}(\hat{o}) \leq \sum_{\boldsymbol{x}\in R_k}\sum_{\boldsymbol{y}\in R_k} \mathrm{tr}\left[|\psi\rangle\langle\psi| A_{\boldsymbol{xy}}\right]. \tag{135}$$

We then have that

$$\mathrm{Var}(\hat{o}) \leq \sum_{\boldsymbol{x}\in R_k}\sum_{\boldsymbol{y}\in R_k} |\mathrm{tr}\left[|\psi\rangle\langle\psi| A_{\boldsymbol{xy}}\right]| \tag{136}$$

$$\leq \sum_{\boldsymbol{x}\in R_k}\sum_{\boldsymbol{y}\in R_k} \frac{e^2(\eta-a-b)!}{(\eta-a)!}. \tag{137}$$

Recall that we defined a and b in Eq. (100) in the following way,

$$a = |\boldsymbol{x} \cap \boldsymbol{y}|, \qquad b = 2k - 2a. \tag{138}$$

Recall also the definition of the set of sequences R_k from Eq. (48),

$$R_k = \{1, \ldots, \eta/k\} \times \{\eta/k + 1, \ldots, 2\eta/k\} \times \cdots \times \{(k-1)\,\eta/k + 1, \ldots, \eta\}\,. \tag{139}$$

Colloquially, a sequence in R_k indexes a set of k registers, one from the first group of η/k, one from the second group of η/k, and so on.

Let us consider a fixed sequence $\boldsymbol{x} \in R_k$ and determine how many sequences $\boldsymbol{y} \in R_k$ exist for a specific value of a. For a fixed value of a, $\boldsymbol{x}$ and $\boldsymbol{y}$ share a elements. By construction, there are $\binom{k}{a}$ different choices for these a elements (because there are k groups and $\boldsymbol{x}$ and $\boldsymbol{y}$ can either match or fail to match in each group). In each of the $k - a$ groups of registers where $\boldsymbol{x}$ and $\boldsymbol{y}$ don't match, there are exactly $\eta/k - 1$ ways to choose the corresponding element of $\boldsymbol{y}$. Therefore, for a given a and $\boldsymbol{x}$, we have that

$$|\{\boldsymbol{y} \in R_k : |\boldsymbol{x} \cap \boldsymbol{y}| = a\}| = \binom{k}{a} (\eta/k - 1)^{k-a}\,. \tag{140}$$

The only way that a particular $\boldsymbol{x}$ or $\boldsymbol{y}$ enters into Eq. (137) is through a and b, so we can use this fact to take the sums over $\boldsymbol{x}$ and $\boldsymbol{y}$, yielding

$$\mathrm{Var}(\hat{o}) \leq \sum_{\boldsymbol{x} \in R_k} \sum_{\boldsymbol{y} \in R_k} \frac{e^2\,(\eta - a - b)!}{(\eta - a)!} \tag{141}$$

$$\leq e^2 \sum_{\boldsymbol{x} \in R_k} \sum_{a=0}^{k} \binom{k}{a} (\eta/k - 1)^{k-a} \frac{(\eta - 2k + a)!}{(\eta - a)!} \tag{142}$$

$$\leq e^2\,(\eta/k)^k \sum_{a=0}^{k} \binom{k}{a} (\eta/k - 1)^{k-a} \frac{(\eta - 2k + a)!}{(\eta - a)!}, \tag{143}$$

under the assumption that $\eta > 2k$ so that we don't have to restrict the sum over a.

Simplifying the inequality further, we find that

$$\mathrm{Var}(\hat{o}) \leq e^2 \left(\eta/k\right)^k \left(\eta/k-1\right)^k \sum_{a=0}^{k} \binom{k}{a} \left(\eta/k-1\right)^{-a} \frac{(\eta-2k+a)!}{(\eta-a)!} \tag{144}$$

$$\leq e^2 \left(\eta/k\right)^k \left(\eta/k-1\right)^k \sum_{a=0}^{k} \binom{k}{a} \left(\eta/k-1\right)^{-a} \frac{(\eta-2k+a)!}{(\eta-k)!}. \tag{145}$$

Now we employ the upper and lower bounds from Stirling's formula (that hold for any integer $n > 0$),

$$\sqrt{2\pi n}\left(\frac{n}{e}\right)^n < n! < e\sqrt{2\pi n}\left(\frac{n}{e}\right)^n. \tag{146}$$

We can use these bounds to simplify the ratio of factorials in Eq. (145),

$$\frac{(\eta-2k+a)!}{(\eta-k)!} \leq e\sqrt{2\pi\left(\eta-2k+a\right)}\left(\frac{\eta-2k+a}{e}\right)^{\eta-2k+a}\frac{1}{(\eta-k)!} \tag{147}$$

$$\leq e\sqrt{2\pi\left(\eta-k\right)}\left(\frac{\eta-k}{e}\right)^{\eta-2k+a}\frac{1}{(\eta-k)!} \tag{148}$$

$$\leq e\left(\frac{\eta-k}{e}\right)^{\eta-2k+a}\left(\frac{e}{\eta-k}\right)^{\eta-k} \tag{149}$$

$$= e\left(\frac{e}{\eta-k}\right)^{k-a}. \tag{150}$$

Using the assumption that $\eta > 2k$ we can proceed further, yielding

$$\frac{(\eta-2k+a)!}{(\eta-k)!} \leq e\left(\frac{e}{\eta-k}\right)^{k-a} \tag{151}$$

$$\leq e\left(\frac{2e}{\eta}\right)^{k-a} \tag{152}$$

$$= e\left(\frac{2e}{\eta}\right)^{k}\left(\frac{2e}{\eta}\right)^{-a}, \tag{153}$$

where we have used the fact that $\eta > 2k$ implies that $\eta - k > \eta/2$.

We can use Eq. (153) to further simplify Eq. (145), finding that,

$$\mathrm{Var}(\hat{o}) \leq e^2 (\eta/k)^k (\eta/k-1)^k \sum_{a=0}^{k} \binom{k}{a} (\eta/k-1)^{-a} \frac{(\eta-2k+a)!}{(\eta-k)!} \tag{154}$$

$$\leq e^3 (\eta/k)^k (\eta/k-1)^k \sum_{a=0}^{k} \binom{k}{a} (\eta/k-1)^{-a} \left(\frac{2e}{\eta}\right)^k \left(\frac{2e}{\eta}\right)^{-a} \tag{155}$$

$$\leq e^3 \left(\eta^2/k^2\right)^k \sum_{a=0}^{k} \binom{k}{a} \left(\frac{\eta}{2k}\right)^{-a} \left(\frac{2e}{\eta}\right)^k \left(\frac{2e}{\eta}\right)^{-a} \tag{156}$$

$$= e^3 \left(\frac{2e\eta}{k^2}\right)^k \sum_{a=0}^{k} \binom{k}{a} \left(\frac{e}{k}\right)^{-a}. \tag{157}$$

Note that we again used the fact that $\eta > 2k$ implies that $\eta - k > \eta/2$ to simplify the part of the bound involving $(\eta/k-1)$. Applying the binomial theorem to the sum yields the bound

$$\mathrm{Var}(\hat{o}) \leq e^3 \left(\frac{2e\eta}{k^2}\right)^k e^{-k} (k+e)^k \tag{158}$$

$$= e^3 \left(\frac{2\eta (k+e)}{k^2}\right)^k. \tag{159}$$

Recall that we defined the estimator $\hat{o}$ by neglecting the coefficient $\frac{k^k(\eta!)}{\eta^k(\eta-k)!}$ in Eq. (49)'s expression for the k-RDM element ${}^k D^{j_1,\ldots,j_k}_{i_1,\ldots,i_k}$. If we let $\hat{d}$ be the estimator for this k-RDM element with the coefficient included, we have that

$$\mathrm{Var}(\hat{d}) = \left(\frac{k^k (\eta!)}{\eta^k (\eta-k)!}\right)^2 \mathrm{Var}(\hat{o}). \tag{160}$$

Therefore, we can bound the desired variance by

$$\mathrm{Var}(\hat{d}) \leq \left(\frac{k^k (\eta!)}{\eta^k (\eta-k)!}\right)^2 e^3 \left(\frac{2\eta (k+e)}{k^2}\right)^k. \tag{161}$$

Simplifying this expression, we obtain

$$\mathrm{Var}(\hat{d}) \leq \left(\frac{k^k (\eta!)}{\eta^k (\eta-k)!}\right)^2 e^3 \left(\frac{2\eta (k+e)}{k^2}\right)^k \tag{162}$$

$$= e^3 \left(\frac{(\eta!)}{(\eta-k)!}\right)^2 \left(\frac{2(k+e)}{\eta}\right)^k \tag{163}$$

$$\leq e^3 \eta^k (2k+2e)^k, \tag{164}$$

which is the bound advertised in Eq. (51).

Supplementary Note 7 More efficient Slater determinant state preparation in first quantization

The general principle is to prepare the state in second quantization, then convert it to first quantization. To avoid needing to store all N qubits for the second-quantized state as it is produced, we convert its qubits to the first-quantized representation.

To explain this, we will first explain how a state in the second-quantized representation can be converted to the first-quantized representation. A computational basis state in second quantization consists of a string of N bits with η ones and $N - \eta$ zeros. The procedure is to run through these qubits in sequence and store the locations in η registers of size $\lceil \log N \rceil$. Let us call the qubit number we consider from the second-quantized representation q and also record the number of electrons (ones) found so far as ξ. The value of ξ will be stored in an ancilla register of size $n_\eta = \lceil \log(\eta + 1) \rceil$.

We initialize all η registers for the first-quantized representation and the ξ register as zero. Then, for $q = 1$ to N we perform the following.

1. Add the value in qubit q to the ξ register, with Toffoli cost $n_\eta - 1$. If the qubit is in the state $|1\rangle$ then ξ is incremented.

2. Now use qubit q to control unary iteration [30] on the register ξ, which has cost $\eta - 1$.

3. Use this unary iteration to write the value q into register ξ using CNOTs. Because q is iterated classically, only CNOTs are needed, with no further Toffolis beyond that needed for the unary iteration. Because the unary iteration is controlled by qubit q, in the case where qubit q is in state $|0\rangle$, the unary iteration does not proceed and the value of q is not written out.

4. Now perform unary iteration on ξ again that is *not* controlled; the cost is $\eta - 2$.

5. We use the unary iteration on ξ to check if the value in register number ξ is q; if it is then we perform a NOT on qubit q. This multiply-controlled Toffoli is controlled by $\lceil \log N \rceil + 1$ qubits (including the qubit from the unary iteration), so it has a cost of $\lceil \log N \rceil$. But, this is done for each of the η registers, for a total cost $\eta \lceil \log N \rceil$.

The last operation ensures that qubit q is set to $|0\rangle$. +That is because, if it is initially $|0\rangle$, then value q is not written in register ξ, and the value is not flipped. If it is initially $|1\rangle$, then q is written in register ξ, and the multiply-controlled Toffoli flips this qubit to $|0\rangle$.

So far this procedure gives an ordered list of the electron positions, but we need an antisymmetrized state. To obtain that, we apply the procedure in [31] to antisymmetrize with cost $\mathcal{O}(\eta \log \eta \log N)$. The total Toffoli cost is

$$N\left(2\eta + n_\eta - 3 + \eta \lceil \log N \rceil\right) + \mathcal{O}(\eta \log \eta \log N). \tag{165}$$

The dominant cost here is $\eta N \log N$ from erasing the qubits in the second-quantized representation, with the factor of $\log N$ coming from the need to check all qubits of each register to check if it is q. However, recall that in unary iteration it is possible to check if a register is equal to a consecutive sequence of values without this logarithmic overhead, and we are considering consecutive values of q.

To eliminate that overhead, we, therefore, consider simultaneous unary iteration on all of the η registers. That is, for each register for the first-quantized representation, we also store the qubits needed for unary iteration, as well as a control register to ensure we do not iterate on registers that do not have value written into them yet. The control qubits will correspond to the value of ξ in unary. Our modified procedure is as follows (with the iteration of q from 1 to N).

1. Perform a single step of unary iteration on all η registers with cost η Toffolis.

2. Add the value in qubit q to the ξ register, with Toffoli cost $n_\eta - 1$.

3. Use qubit q to control unary iteration on the register ξ, which has cost $\eta - 1$.

4. Use this unary iteration to write the value q into register ξ, *as well* as the $\lceil \log N \rceil$ ancilla qubits for the unary iteration and the control qubit. Again this is performed with CNOTs.

5. Convert the control qubits to one-hot unary using a sequence of CNOTs.

6. For each of the η registers, use the control qubit and the unary iteration output to control a NOT on qubit q. This has a cost of a single Toffoli for each register, for a toal of η.

7. Convert the control qubits to from one-hot unary with CNOTs.

As a result, we have eliminated the $\log N$ factor and also eliminated the cost of $\eta - 2$ for the unary iteration on ξ (because the control qubits are a unary representation of ξ). One might ask if the binary representation of ξ is still needed; however, it would be more costly to add increment ξ in unary (about η cost instead of $\log \eta$). The total Toffoli cost of this procedure is now

$$N\left(3\eta + n_\eta - 2\right) + \mathcal{O}(\eta \log \eta \log N), \tag{166}$$

where the order term is the cost for antisymmetrizing. Note that this reduces the Toffoli cost, but there is still a Clifford cost of $N\eta \log N$ from the CNOTs to place the value of q in the first-quantized registers.

Now to efficiently prepare the Slater determinant, we can perform the sequence of Givens rotations on the qubits for the second-quantized representation. The Givens rotations are performed in a sequence where Givens rotations are performed in a layer on qubits 1 to $\eta + 1$, then on qubits 2 to $\eta + 2$, then 3 to $\eta + 3$, and so on. One can find the details of the Givens rotations that must be applied in [32]. Generally, layer q of Givens rotations is performed on qubits q to $\eta + q$. After the first layer there are only $\eta + 1$ qubits being used, and the first qubit is not accessed

again in the preparation. Therefore we can convert this qubit to the first-quantized representation and erase it. Then there are only η qubits actively being used in the second-quantized representation, and the next layer will be performed on qubits 2 to $\eta + 2$, bringing on one more qubit.

In this way, each time we perform a layer of Givens rotations to prepare the state, we can convert one qubit to the first-quantized representation, and only $\eta + 1$ qubits of the second-quantized representation need be used at once, which is trivial compared to the number of qubits used for the first-quantized representation.

References and Notes

[1] Low, G. H., Su, Y., Tong, Y. & Tran, M. C. On the complexity of implementing Trotter steps (2022). URL `https://arxiv.org/abs/2211.09133`.

[2] Kassal, I., Jordan, S. P., Love, P. J., Mohseni, M. & Aspuru-Guzik, A. Polynomial-time quantum algorithm for the simulation of chemical dynamics. *Proceedings of the National Academy of Sciences* **105**, 18681–18686 (2008). URL `http://www.pnas.org/content/105/48/18681.abstract`.

[3] Chan, H. H. S., Meister, R., Jones, T., Tew, D. P. & Benjamin, S. C. Grid-based methods for chemistry simulations on a quantum computer (2022). URL `https://arxiv.org/abs/2202.05864`.

[4] Cody Jones, N. *et al.* Faster quantum chemistry simulation on fault-tolerant quantum computers. *New Journal of Physics* **14**, 115023 (2012). URL `http://stacks.iop.org/1367-2630/14/i=11/a=115023?key=crossref.51339e3e7dfc030625bd8da9496cc34b`.

[5] Babbush, R., Berry, D. W., McClean, J. R. & Neven, H. Quantum Simulation of Chem-

istry with Sublinear Scaling in Basis Size. *npj Quantum Information* **5**, 92 (2019). URL https://www.nature.com/articles/s41534-019-0199-y.

[6] Su, Y., Berry, D., Wiebe, N., Rubin, N. & Babbush, R. Fault-tolerant quantum simulations of chemistry in first quantization. *PRX Quantum* **4**, 040332 (2021). URL https://journals.aps.org/prxquantum/abstract/10.1103/PRXQuantum.2.040332.

[7] Kivlichan, I. D., Wiebe, N., Babbush, R. & Aspuru-Guzik, A. Bounding the costs of quantum simulation of many-body physics in real space. *Journal of Physics A: Mathematical and Theoretical* **50**, 305301 (2017). URL http://iopscience.iop.org/article/10.1088/1751-8121/aa77b8.

[8] Childs, A. M., Leng, J., Li, T., Liu, J.-P. & Zhang, C. Quantum Simulation of Real-Space Dynamics. *arXiv:2203.17006* (2022). URL https://arxiv.org/abs/2203.17006.

[9] Chen, Y.-G. & Weeks, J. D. Local molecular field theory for effective attractions between like charged objects in systems with strong Coulomb interactions. *Proceedings of the National Academy of Sciences* **103**, 7560–7565 (2006).

[10] González-Espinoza, C. E., Ayers, P. W., Karwowski, J. & Savin, A. Smooth models for the Coulomb potential. *Theoretical Chemistry Accounts* **135**, 256 (2016).

[11] Manzer, S., Horn, P. R., Mardirossian, N. & Head-Gordon, M. Fast, accurate evaluation of exact exchange: The occ-RI-K algorithm. *J. Chem. Phys.* **143**, 024113 (2015).

[12] Lin, L. Adaptively Compressed Exchange Operator. *J. Chem. Theory Comput.* **12**, 2242–2249 (2016).

[13] Rokhlin, V. Rapid solution of integral equations of classical potential theory. *Journal of Computational Physics* **60**, 187–207 (1985).

[14] Barnes, J. & Hut, P. A hierarchical O(N log N) force-calculation algorithm. *Nature* **324**, 446–449 (1986).

[15] Darden, T., York, D. & Pedersen, L. Particle mesh Ewald: An N log N method for Ewald sums in large systems. *The Journal of Chemical Physics* **98**, 10089–10092 (1993). URL `http://aip.scitation.org/doi/10.1063/1.464397`.

[16] Low, G. H. & Wiebe, N. Hamiltonian Simulation in the Interaction Picture. *arXiv:1805.00675* (2018). URL `http://arxiv.org/abs/1805.00675`.

[17] Carrier, J., Greengard, L. & Rokhlin, V. A Fast Adaptive Multipole Algorithm for Particle Simulations. *SIAM Journal on Scientific and Statistical Computing* **9**, 669–686 (1988).

[18] Huang, H.-Y., Kueng, R. & Preskill, J. Predicting many properties of a quantum system from very few measurements. *Nature Physics* **16**, 1050–1057 (2020).

[19] Zhao, A., Rubin, N. C. & Miyake, A. Fermionic Partial Tomography via Classical Shadows. *Physical Review Letters* **127**, 110504 (2021). URL `https://link.aps.org/doi/10.1103/PhysRevLett.127.110504`.

[20] Wan, K., Huggins, W. J., Lee, J. & Babbush, R. Matchgate Shadows for Fermionic Quantum Simulation. *arXiv:2207.13723* (2022). URL `http://arxiv.org/abs/2207.13723`.

[21] O'Gorman, B. Fermionic tomography and learning. *arXiv:2207.14787* (2022). URL `http://arxiv.org/abs/2207.14787`.

[22] Low, G. H. Classical shadows of fermions with particle number symmetry. *arXiv:2208.08964* (2022). URL https://arxiv.org/abs/2208.08964.

[23] Bravyi, S. & Maslov, D. Hadamard-free circuits expose the structure of the clifford group. *IEEE Trans. Inf. Theory* **67**, 4546–4563 (2021). URL https://ieeexplore.ieee.org/document/9435351/.

[24] Huggins, W. J. *et al.* Unbiasing fermionic quantum monte carlo with a quantum computer. *Nature* **603**, 416–420 (2022). URL http://dx.doi.org/10.1038/s41586-021-04351-z. 2106.16235.

[25] Lerasle, M. Lecture notes: Selected topics on robust statistical learning theory. *arXiv:1908.10761* (2019). URL http://arxiv.org/abs/1908.10761.

[26] García, H. J., Markov, I. L. & Cross, A. W. On the geometry of stabilizer states. *arXiv:1711.07848* (2017). URL http://arxiv.org/abs/1711.07848.

[27] Aaronson, S. & Gottesman, D. Improved simulation of stabilizer circuits. *Phys. Rev. A* **70** (2004). URL https://link.aps.org/doi/10.1103/PhysRevA.70.052328.

[28] Gottesman, D. The heisenberg representation of quantum computers. Tech. Rep. LA-UR-98-2848; CONF-980788-, Los Alamos National Lab., NM (United States) (1998). URL https://www.osti.gov/biblio/319738-heisenberg-representation-quantum-computers.

[29] Gross, D., Krahmer, F. & Kueng, R. A partial derandomization of PhaseLift using spherical designs. *J. Fourier Anal. Appl.* **21**, 229–266 (2015). URL http://link.springer.com/10.1007/s00041-014-9361-2.

[30] Babbush, R. *et al.* Encoding Electronic Spectra in Quantum Circuits with Linear T Complexity. *Physical Review X* **8**, 041015 (2018). URL `https://journals.aps.org/prx/abstract/10.1103/PhysRevX.8.041015`.

[31] Berry, D. W. *et al.* Improved Techniques for Preparing Eigenstates of Fermionic Hamiltonians. *npj Quantum Information* **4**, 22 (2018). URL `http://www.nature.com/articles/s41534-018-0071-5`.

[32] Kivlichan, I. *et al.* Quantum Simulation of Electronic Structure with Linear Depth and Connectivity. *Physical Review Letters* **120** (2018).
