# Supplementary material for: Quantum simulation of exact electron dynamics can be more efficient than classical mean-field methods
Source: Nat Commun. 2023 Jul 10;14:4058. doi: 10.1038/s41467-023-39024-0 (PMC10333266; doi:10.1038/s41467-023-39024-0)
Supplement: Supplementary file 2 — Peer Review [file 41467_2023_39024_MOESM2_ESM.pdf]

nature portfolio

Peer Review File

Quantum simulation of exact electron dynamics can be more efficient than classical mean-field methods

Open Access This file is licensed under a Creative Commons Attribution 4.0 International License, which permits use, sharing, adaptation, distribution and reproduction in any medium or format, as long as you give appropriate credit to the original author(s) and the source, provide a link to the Creative Commons license, and indicate if changes were made. In the cases where the authors are anonymous, such as is the case for the reports of anonymous peer reviewers, author attribution should be to 'Anonymous Referee' followed by a clear attribution to the source work. The images or other third party material in this file are included in the article's Creative Commons license, unless indicated otherwise in a credit line to the material. If material is not included in the article's Creative Commons license and your intended use is not permitted by statutory regulation or exceeds the permitted use, you will need to obtain permission directly from the copyright holder. To view a copy of this license, visit http://creativecommons.org/licenses/by/4.0/.

REVIEWER COMMENTS

Reviewer #1 (Remarks to the Author):

Babbush et al. show that quantum computing algorithms may be more scalable than mean-field quantum chemistry algorithms on classical computers. In particular, they analyze the number of operations and memory required for time-evolution updates of electron dynamics simulations. Their work rigorously disproves that quantum computing is only useful where high levels of their are typically employed on classical computers. Rather, it is generally applicable to molecules independent of their electron correlation.

This article provides an important and original viewpoint for the application of quantum computers to quantum chemistry. It is well written and for the most part it is easy to follow. For these reasons, I recommend publication in Nature Communications after addressing a few minor comments given below.

The article focuses primarily on the computational cost of propagating an electronic wavefunction in time, which begs the question whether similar arguments could be made for static mean-field calculations. The arguments made in the paper could in principle be applied to imaginary-time eigensolvers (for example, https://doi.org/10.1103/PhysRevResearch.4.033121) in comparison to established mean-field eigensolvers on classical computers. Of course, this may not be a straightforward comparison since efficient classical eigensolvers do not use time evolution.

Other minor comments:
- Under Eq. 4, det(C_occ) is not the Slater determinant. C_occ is an $\eta \times N$ matrix and does not in general have a determinant. In this case the Slater determinant is the determinant of the matrix of η particles in η occupied orbitals.
- The error tolerance ε is used below Eq. 4, but it is not defined until the text above Eq. 10.
- The notation in the appendices needs to be carefully proofread. Some variables such as script-V for the potential operator and σ for a density operator do not appear to be defined in the text.

Reviewer #2 (Remarks to the Author):

This manuscript provides in-depth review and analysis of classical mean-field methods and exact quantum algorithms for electron dynamics. The authors cover the state-of-the-art quantum simulation algorithms with consideration of the different choice of basis functions. The overheads on state preparation and measuring quantities in quantum algorithms are taken into account, for which the authors introduce new stragies for initializing Slater determinants and measuring reduced density matrix via classical shadows. Finally, they classify diverse polynomial power of quantum speedups with respect to the tradeoff of the number of basis functions and particles.

I think this work includes noteworthy methodologies and results in the design and analysis of quantum algorithms. The work is of significant to quantum simulation and chemical mean-field simulation, towards better comprehension of practical quantum advantage in quantum chemistry. I recommend acceptance for this work after appropriately addressing some minor issues below.

* Explain the meaning of "exact" in the beginning.

* In Discussion, the power of quantum speedups (seventh， quartic，super-quadratic，quintic) in various regimes are not so clear enough, and the explanation in Appendix E appears to be lengthy. It is better to point out these powers in Table I.

* In Appendix D, the error in smoothing Coulumb operator in real-space should be considered. Should we choose V_max = O(1/\epsilon), which adds an additional 1/\epsilon factor?

Reviewer #3 (Remarks to the Author):

Quantum simulation is one of the most important applications of quantum computing. In the near term, quantum simulation of chemistry models is promising, and there has been systematic study between simulation by quantum algorithms and classical numerical methods. This submission falls into this category. In particular, this paper tightens the quantum complexity bounds of certain first quantized quantum algorithms, which take exponentially less space and polynomially fewer operations. This is achieved by leveraging recent techniques for bounding Trotter error. Compared to the classical algorithms by Hartree-Fock and density functional theory, the proposed quantum algorithms are more efficient.

From my perspective, the paper is a solid work on the topic on theoretical aspects of quantum chemistry and quantum algorithms. It is able to combine up-to-date algorithm design and analysis into the simulation of exact electron dynamics, and it proved clearly the sublinear gate complexity using Trotter based methods (Appendix B), calculated the constant factors in the interaction-picture plane-wave algorithm (Appendix C), made a clear comparison of the game complexity and speedup in various regimes (Appendix E), etc.

However, to me Nature Communications calls for papers with significant novelty and general impact. Those are the notable weaknesses of the current submission:

- The technical contributions are a bit incremental. Appendix B heavily relies on Ref. [42], Appendix C heavily relies on Ref. [48], Appendix F heavily relies on Ref. [57]. From the high-level perspective of quantum simulation algorithms for chemistry, the paper is more like applying up-to-date tools in a correct way and combine them to give new results. This is a not fault per se, if the problem it solved is well-established and very important. However, another fact is:

- The regime where the proposed quantum algorithms work well is a bit subtle. Figure 1 clearly shapes the range where the algorithm achieves super-quadratic quantum speedup: when alpha<5/4 and when alpha>4; here N=\Theta(\eta^{\alpha}) is the power of \eta of N. It’s not so clear what this means – which electron models in practice satisfy this parameter range? It would be much more helpful if the connection between the parameter range and practical models is articulated clearly.

Given these two points above, my personal take is that the authors understand relevant up-to-date techniques in quantum algorithms and quantum chemistry very well, and are able to combine them to prove new results in simulating electron dynamical models. This is good to know and the paper deserves to get published in a decent venue. However, given the high standard of Nature Communications, the novelty of technical contributions is not significant enough, and the scenario that the proposed quantum algorithm being very helpful is a bit opaque. In all, I cannot recommend acceptance for this paper to Nature Communications.

Suggestions:

- In general, I found the main body of the paper hard to digest – there are only sparse formulas, tables, one table, but huge chunk of discussions in English words. This is very hard to follow. In general, the main body reads more like an extended abstract of a systematic study, not a self-contained appealing scientific article (that many Nature Communications papers enjoy). There are also many places where the expressions require further clarification, such as “Under certain assumptions, the norm of the potential term can be reduced to a polylogarithmic dependence on N (see Appendix D for more details)“ and “While certain aspects of these systems are conspicuously classical, they still present spectra that can be challenging to model [82, 83].”

It would be helpful if the main body can be more streamlined, articulating the technical contribution,

and articulating the proved results. For instance, I personally found Table II and Figure 1 very helpful; maybe the authors can move them to the main body.

- Is it possible to conduct some experiments, at least some numerical experiments simulated on classical computers? I personally always found claims about quantum algorithms better than the classical counterparts for quantum chemistry and quantum many-body physics a bit overselling, because many classical empirical tools have been widely applied with good performance, such as DMRG. As the title of the paper directly claims that their quantum algorithms are more efficient than classical mean-field methods, I feel that evidence from a practical perspective can be helpful.

- Typo: Between (F2) and (F3), Ref. 57 -> Ref. [57]. This typo applies to many later places also. (I think in this template, references should be embraced by brackets.)

Reviewer #1 (Remarks to the Author):

Babbush et al. show that quantum computing algorithms may be more scalable than mean-field quantum chemistry algorithms on classical computers. In particular, they analyze the number of operations and memory required for time-evolution updates of electron dynamics simulations. Their work rigorously disproves that quantum computing is only useful where high levels of their are typically employed on classical computers. Rather, it is generally applicable to molecules independent of their electron correlation.

This article provides an important and original viewpoint for the application of quantum computers to quantum chemistry. It is well written and for the most part it is easy to follow. For these reasons, I recommend publication in Nature Communications after addressing a few minor comments given below.

We are very glad to hear that the reviewer appreciates the contribution of our work and recommends publication in Nature Communications!

The article focuses primarily on the computational cost of propagating an electronic wavefunction in time, which begs the question whether similar arguments could be made for static mean-field calculations.

We thank the referee for this comment. This is an interesting idea and several of our findings might be helpful for establishing such a result. However, significant additional challenges beyond the scope of our work would need to be overcome to formulate a quantum algorithm that gives a speedup for solving the static mean-field problem. For example, one would need a method of computing the Fock operator on-the-fly using the quantum computer using the current estimate of the lowest energy occupied orbitals. Also, a self-consistent field approach to the static mean-field problem is nonlinear and thus it is unclear (at least to us) how to perform this as a straightforward unitary circuit.

The arguments made in the paper could in principle be applied to imaginary-time eigensolvers (for example, https://doi.org/10.1103/PhysRevResearch.4.033121) in comparison to established mean-field eigensolvers on classical computers. Of course, this may not be a straightforward comparison since efficient classical eigensolvers do not use time evolution.

We thank the referee for this comment. We indeed thought about the application of our technique in the context of imaginary time evolution. We would like to point out that the reason we focus on real-time dynamics is that it circumvents any cost that scales with any quantities that grow exponentially with system size or time. On the contrary, any thermal state preparation (i.e., imaginary time evolution) algorithms have a cost that scales exponentially with system size and imaginary time (i.e., inverse temperature.) This applies to the PITE algorithm proposed in the paper cited by the referee. Due to this reason, we did not attempt to perform a precise cost analysis of imaginary-time evolution using the first-quantization techniques developed in this work.

Other minor comments:
- Under Eq. 4, det(C_occ) is not the Slater determinant. C_occ is an η×N matrix and does not in general have a determinant. In this case the Slater determinant is the determinant of the matrix of η particles in η occupied orbitals.

We thank the referee for pointing this mistake out. It was indeed a typo, and we modified the corresponding text accordingly.

- The error tolerance ε is used below Eq. 4, but it is not defined until the text above Eq. 10.

We thank the referee for pointing this out. We have updated our manuscript to state clearly the definition of epsilon as early as the introduction. Note that several slightly different epsilons appear throughout the paper but they are all asymptotically equivalent and only appear in asymptotic scalings so it should be okay.

- The notation in the appendices needs to be carefully proofread. Some variables such as script-V for the potential operator and σ for a density operator do not appear to be defined in the text.

We thank the referee for this comment. We have made sure to define these quantities more clearly in the new version of the text.

Reviewer #2 (Remarks to the Author):

This manuscript provides in-depth review and analysis of classical mean-field methods and exact quantum algorithms for electron dynamics. The authors cover the state-of-the-art quantum simulation algorithms with consideration of the different choice of basis functions. The overheads on state preparation and measuring quantities in quantum algorithms are taken into account, for which the authors introduce new strategies for initializing Slater determinants and measuring reduced density matrix via classical shadows. Finally, they classify diverse polynomial power of quantum speedups with respect to the tradeoff of the number of basis functions and particles.

I think this work includes noteworthy methodologies and results in the design and analysis of quantum algorithms. The work is of significant to quantum simulation and chemical mean-field simulation, towards better comprehension of practical quantum advantage in quantum chemistry. I recommend acceptance for this work after appropriately addressing some minor issues below.

We thank the referee for their positive assessment of our work and recommendation of publication subject to minor changes.

* Explain the meaning of "exact" in the beginning.

We thank the referee for this comment. We have added a sentence to the first paragraph of the introduction which addresses exactly this.

* In Discussion, the power of quantum speedups (seventh, quartic, super-quadratic, quintic) in various regimes are not so clear enough, and the explanation in Appendix E appears to be lengthy. It is better to point out these powers in Table I.

We thank the referee for this comment. Based on this feedback and comments of other reviewers we have elected to move Figure 1 into the main body and simply refer the reader to that figure in favor of the (prior) slightly confusing verbal explanation of the speedup powers. This gives a more holistic picture of the speedup order by collapsing the entire cost to a single parameter as opposed to both eta and N.

* In Appendix D, the error in smoothing Coulumb operator in real-space should be considered. Should we choose V_max = O(1/\epsilon), which adds an additional 1/\epsilon factor?

We thank the referee for this excellent comment. The referee is correct that smoothing the Coulomb operator in real space introduces additional errors. If done in the most naive way then something like the scaling the reviewer suggests is correct. However, there are better strategies than this that involve using extrapolation schemes. We try to be clear about this in Appendix D by writing:

Using such a drastic cutoff will introduce a significant bias into the overall dynamics. In order to avoid this, papers such as [88,89] have sought to develop Richardson extrapolation type schemes where simulations are run with a series of smoothing or cutoff parameters in order to extrapolate the value of the observable with zero cutoff. However, questions remain about the convergence of such procedures and it seems likely to re-introduce some polynomial dependence on N in order to reach convergence with the continuum limit. Nevertheless, the context of this paper is that one might be interested in getting a speedup over low accuracy classical algorithms. In that spirit, one could probably make the case that if merely trying to improve in speed over mean-field algorithms, the error introduced in imposing a cutoff in the Coulomb operator might be less significant than the error due to making the mean-field approximation. Thus, this is perhaps a valid approach when competing with such classical methods, and thus might provide an exponential speedup.

We do not know exactly how this approach scales in terms of epsilon, although it is likely better than 1/epsilon. Due to this uncertainty, we mention this approach in Appendix D and in one sentence in the middle of the paper but we do not claim this as a result in our abstract, intro, tables, or discussion. We do feel it is worth mentioning though since it is the only way to compare to certain prior scalings claimed in works such as [54].

Reviewer #3 (Remarks to the Author):

Quantum simulation is one of the most important applications of quantum computing. In the near term, quantum simulation of chemistry models is promising, and there has been systematic study between simulation by quantum algorithms and classical numerical methods. This submission falls into this category. In particular, this paper tightens the quantum complexity bounds of certain first quantized quantum algorithms, which take exponentially less space and polynomially fewer operations. This is achieved by leveraging recent techniques for bounding Trotter error. Compared to the classical algorithms by Hartree-Fock and density functional theory, the proposed quantum algorithms are more efficient.

From my perspective, the paper is a solid work on the topic on theoretical aspects of quantum chemistry and quantum algorithms. It is able to combine up-to-date algorithm design and analysis into the simulation of exact electron dynamics, and it proved clearly the sublinear gate complexity using Trotter based methods (Appendix B), calculated the constant factors in the interaction-picture plane-wave algorithm (Appendix C), made a clear comparison of the game complexity and speedup in various regimes (Appendix E), etc.

We are glad that the referee appreciates these contributions!

However, to me Nature Communications calls for papers with significant novelty and general impact. Those are the notable weaknesses of the current submission:

- The technical contributions are a bit incremental. Appendix B heavily relies on Ref. [42], Appendix C heavily relies on Ref. [48], Appendix F heavily relies on Ref. [57]. From the high-level perspective of quantum simulation algorithms for chemistry, the paper is more like applying up-to-date tools in a correct way and combine them to give new results. This is a not fault per se, if the problem it solved is well-established and very important.

We thank the referee for this question.

The point of Appendix B is essentially to show that the results of Ref. [42] extend to first quantization. The purpose is to show how and why it is possible to use [42] in our context. We agree this is not particularly challenging, but it results in a very significant reduction in Trotter errors over what was previously known for first quantization, giving what is the best scaling quantum algorithm for chemistry in many contexts.

We agree Appendix C is an incremental result based on Ref. [48]; it is included for completeness.

We do not think Appendix F is incremental, and we believe this required a substantial innovation of our own. Most previous work used exponentially more circuit repetitions (in N) to accurately characterize fermionic k-RDMs. Even the very recent Ref. [61], which achieves a scaling that is

logarithmic in N, requires exponentially more classical post processing (in N) to estimate an element of the k-RDM than our proposed approach. The classical shadows protocol that we introduce uses similar concepts as the first work on classical shadows [57], which has resulted into many related follow-up pulbications in venues like Nature. Contrary to the original classical shadow approach, taking advantage of the antisymmetry of the wavefunction in first quantization demands entirely different analysis techniques and require a significant amount of work and ideas as shown in Appendix F.

These comments also leave out many of the valuable contributions of our work. For example, Appendix A and associated discussion in the manuscript which establishes formal bounds on the cost of classical mean-field methods. No such analysis had been performed previously and allows us to compare quantum algorithms to classical mean-field algorithms on an even footing. There is then also Appendix G and the associated discussion describing those results in the main body which gives a very significant speedup (from O(eta^2 N) to O(eta N)) in the cost of mean-field state preparation. The technique for this is quite involved.

Most importantly, there is the main finding of our paper, which emerges from the sum of our contributions: that quantum algorithms can be more efficient than even the most efficient and inaccurate classical algorithms (e.g., when entanglement is not required for accurate simulation, as in mean-field systems). We believe this finding is transformative as it suggests that broad classes of systems not previously considered as candidates for quantum speedups may yet see a quantum speedup after all. It opens up many new places to look for applications.

However, another fact is:

- The regime where the proposed quantum algorithms work well is a bit subtle. Figure 1 clearly shapes the range where the algorithm achieves super-quadratic quantum speedup: when alpha<5/4 and when alpha>4; here N=\Theta(\eta^{\alpha}) is the power of \eta of N. It's not so clear what this means – which electron models in practice satisfy this parameter range? It would be much more helpful if the connection between the parameter range and practical models is articulated clearly.

The proposed quantum algorithms work in all regimes and there is always a quantum speedup, as shown in Figure 1. We maintain that it is quite surprising and interesting that there is ever a quantum speedup (even if sub-quadratic) when simulating systems that do not require entanglement for their description (e.g., mean-field systems). We believe this is already a significant finding.

But indeed, for the most practical impact, super-quadratic speedups are of interest since they are more likely to overcome the high costs of error-correction on the first generation of such devices, and there are only some parameter regimes for which that is true. The most coarse grained models will fall into the \alpha < 5/4 super-quadratic regime and the most accurate models will fall into the \alpha > 4 super-quadratic regime.

We also want to emphasize that a comparison to zero temperature mean-field simulations is, in this context, the cheapest simulation for a classical computer. As we emphasize in our discussion section, if the simulation is at finite temperature, or if it is a model requiring anything more than mean-field treatment, then the speedup is appreciably larger (more than quadratic in all regimes). So these findings are expected to be impactful in practice.

Given these two points above, my personal take is that the authors understand relevant up-to-date techniques in quantum algorithms and quantum chemistry very well, and are able to combine them to prove new results in simulating electron dynamical models. This is good to know and the paper deserves to get published in a decent venue.

We are glad that the referee appreciates our findings and believes they are appropriate for publication in a decent venue.

However, given the high standard of Nature Communications, the novelty of technical contributions is not significant enough, and the scenario that the proposed quantum algorithm being very helpful is a bit opaque. In all, I cannot recommend acceptance for this paper to Nature Communications.

As mentioned above, we want to emphasize that our work contains several significant findings and new techniques: (1) the first tight bounds on the number of steps required for classical mean-field dynamics, (2) tighter bounds on the cost of first quantized Trotter approaches giving the best known approach for simulating chemistry across a broad range of parameters, (3) a new approach to measuring k-particle RDMs (including for k=1, which completely characterizes mean-field states) with exponentially reduced sample complexity in N over prior methods, and (4) new state preparation techniques that reduce mean-field state prep from O(eta^2 N) to O(eta N).

We argue that these contributions support the following highly surprising and important finding (5): that quantum algorithms often give a significant speedup over even classical systems that are well treated at mean-field level of theory. We then argue that (6) finite temperature systems, and those requiring more correlation, realize a strictly greater speedup.

We believe these findings and the very conclusion of this work are of high interest to a broad audience and that *Nature Communications* is an appropriate place for it.

Suggestions:

- In general, I found the main body of the paper hard to digest – there are only sparse formulas, tables, one table, but huge chunk of discussions in English words. This is very hard to follow. In general, the main body reads more like an extended abstract of a systematic study, not a self-contained appealing scientific article (that many Nature Communications papers enjoy).

We agree that the paper relies somewhat more on exposition than many works performing theoretical computer science analysis. And indeed, we do relegate many of the most technical proof details to our supplement. However, we did this deliberately in order to make the paper more accessible to the broad audience of *Nature Communications*. We made an attempt to describe the high level content of all of these proofs in the main body. We would also like to note that other reviewers positively commented on the general readability of our manuscript.

There are also many places where the expressions require further clarification, such as “Under certain assumptions, the norm of the potential term can be reduced to a polylogarithmic dependence on N (see Appendix D for more details)“

There are significant length restrictions in *Nature Communications* and this is a relatively minor technical detail rather than something claimed as a main result. We are running up against those limits and have needed to cut content from our main body to address the reviewer’s suggestion to move Figure 1 into the main body. Thus, the best solution that we came up with is to refer people to our more detailed discussion in the Appendix. If the referee has a suggestion to address this point, we would be very happy to accommodate the suggestion. Furthermore, if this is really a sticking point, it would be possible for us to remove Appendix D as this is not really our main result.

and “While certain aspects of these systems are conspicuously classical, they still present spectra that can be challenging to model [82, 83].”

We thank the referee for this comment. Indeed, more details seem appropriate to clarify the corresponding sentence. For HDMs, there is still room for theoretical developments beyond what is classically done and as an example we wanted to point out the currently elusive opacity of matters at stellar interior temperatures.

It would be helpful if the main body can be more streamlined, articulating the technical contribution, and articulating the proved results. For instance, I personally found Table II and Figure 1 very helpful; maybe the authors can move them to the main body.

We thank the referee for this suggestion. This is a good suggestion that was also flagged by another reviewer. We have streamlined the discussion by adding Figure 1 (and an associated discussion) to the main body.

- Is it possible to conduct some experiments, at least some numerical experiments simulated on classical computers? I personally always found claims about quantum algorithms better than the classical counterparts for quantum chemistry and quantum many-body physics a bit overselling, because many classical empirical tools have been widely applied with good performance, such as DMRG. As the title of the paper directly claims that their quantum algorithms are more efficient than classical mean-field methods, I feel that evidence from a practical perspective can be helpful.

We thank the referee for this question. Our paper contains proofs on the scaling of these classical methods. Numerical simulations would perhaps help to clarify the constant factors in the classical scaling. But without knowing the constant factors in the scaling of quantum methods, it is unclear what would be gained in terms of a comparison. It would require very significant further analysis (an entirely new project) to determine the constants in the scaling of the quantum algorithms. For example, the constant factors in the number of Trotter steps required in this representation is unknown and difficult to estimate numerically. Other classical methods that we did not analyze in this paper are only more costly with a worse scaling than the classical methods we compare to in our paper (mean-field methods). Thus, our quantum algorithms would have large polynomial speedups over such methods in all regimes. This, therefore, does not affect the main conclusion of our paper and only strengthens the conclusion of the paper. We do note this aspect in the conclusion of our paper.

- Typo: Between (F2) and (F3), Ref. 57 -> Ref. [57]. This typo applies to many later places also. (I think in this template, references should be embraced by brackets.)

We thank referee for pointing out this typo. We have made this change.

REVIEWER COMMENTS

Reviewer #2 (Remarks to the Author):

The authors have suitably handled my comments. I would like to recommend acceptance.

Reviewer #3 (Remarks to the Author):

I would like to thank the authors for the detailed replies and careful revision of the manuscript. This version is better than the initial version. However, I still have the following questions/suggestions, with "…" quoting parts the authors' reply in the rebuttal letter:

--- "We do not think Appendix F is incremental, and we believe this required a substantial innovation of our own. Most previous work used exponentially more circuit repetitions (in N) to accurately characterize fermionic k-RDMs. Even the very recent Ref. [61], which achieves a scaling that is logarithmic in N, requires exponentially more classical post processing (in N) to estimate an element of the k-RDM than our proposed approach. The classical shadows protocol that we introduce uses similar concepts as the first work on classical shadows [57], which has resulted into many related follow-up publications in venues like Nature. Contrary to the original classical shadow approach, taking advantage of the antisymmetry of the wavefunction in first quantization demands entirely different analysis techniques and require a significant amount of work and ideas as shown in Appendix F."

My reply: I see, thanks for the argument – it is a good point that Ref. [61] requires exponentially more classical post processing time. Could you articulate the difference, and the novelty of your work, at the beginning or end of Appendix F?

--- "These comments also leave out many of the valuable contributions of our work. For example, Appendix A and associated discussion in the manuscript which establishes formal bounds on the cost of classical mean-field methods. No such analysis had been performed previously and allows us to compare quantum algorithms to classical mean-field algorithms on an even footing."

My reply: Regarding Appendix A, could you write a formal theorem statement with a formal proof for the scaling bound in (A14) and (A15)? Currently, the argument is not as formal as I expected. For instance, between (A10) and (A11), it simply says that "with neighboring grid points separated by δ, the smallest the average separation can be is O(η^{1/3}δ)". Why is it this specific value? I agree that norms and scaling bounds are interesting, but for the quality of Nature Communications, it would be better to present a rigorous proof here.

--- "The proposed quantum algorithms work in all regimes and there is always a quantum speedup, as shown in Figure 1. We maintain that it is quite surprising and interesting that there is ever a quantum speedup (even if sub-quadratic) when simulating systems that do not require entanglement for their description (e.g., mean-field systems). We believe this is already a significant finding."

My reply: I'm not sure if I'm following this – I think in Appendix B, you need to know the parameters in the first quantization when you apply Trotter based methods? It would be helpful if you could articulate clearly what description you require for your quantum algorithms.

--- "We also want to emphasize that a comparison to zero temperature mean-field simulations is, in this context, the cheapest simulation for a classical computer. As we emphasize in our discussion section, if the simulation is at finite temperature, or if it is a model requiring anything more than mean-field treatment, then the speedup is appreciably larger (more than quadratic in all regimes). So these findings are expected to be impactful in practice."

My reply: I feel that there is a gap here – which practical models enjoy the parameter ranges with more than quadratic speedup? Previously I asked “It would be much more helpful if the connection between the parameter range and practical models is articulated clearly”, and it would be helpful that the connection to practical models is better explained.

--- “There are significant length restrictions in Nature Communications and this is a relatively minor technical detail rather than something claimed as a main result. We are running up against those limits and have needed to cut content from our main body to address the reviewer’s suggestion to move Figure 1 into the main body. Thus, the best solution that we came up with is to refer people to our more detailed discussion in the Appendix. If the referee has a suggestion to address this point, we would be very happy to accommodate the suggestion. Furthermore, if this is really a sticking point, it would be possible for us to remove Appendix D as this is not really our main result.”

My reply: I understand that Nature Communications have a tight page limit, but this is not a reason to have a storyline not compelling enough and on the other hand have technical many appendices. The page-limit mechanism, to me, picks neat results that can be digested in a few pages. The technical contributions in the appendices of this manuscript are definitely interesting, but there’s simply no space for all of them.

Regarding suggestions, if possible, it might be reasonable to shorten the section “Classical mean-field dynamics” a bit. It currently takes about two pages talking about bounds from different algorithms. This is probably up to the authors’ discretion, but I’m not sure if all of them shall be expanded here and maybe some parts can be a bit briefer. After all, the main contribution is on the quantum computing side.

Appendix D is actually okay to me, after all it’s not long. I think the main point is to better present the storyline in the main body and make it cleaner.

--- “Our paper contains proofs on the scaling of these classical methods. Numerical simulations would perhaps help to clarify the constant factors in the classical scaling. But without knowing the constant factors in the scaling of quantum methods, it is unclear what would be gained in terms of a comparison. It would require very significant further analysis (an entirely new project) to determine the constants in the scaling of the quantum algorithms. For example, the constant factors in the number of Trotter steps required in this representation is unknown and difficult to estimate numerically. Other classical methods that we did not analyze in this paper are only more costly with a worse scaling than the classical methods we compare to in our paper (mean-field methods). Thus, our quantum algorithms would have large polynomial speedups over such methods in all regimes. This, therefore, does not affect the main conclusion of our paper and only strengthens the conclusion of the paper. We do note this aspect in the conclusion of our paper.”

My reply: I very much agree that it requires significant further effort to determine the constants in the scaling of the quantum algorithms. Nevertheless, maybe the authors can at least make a bit more efforts and let the readers know where the potential overheads can come from? This reply mentions the constant factors in the number of Trotter steps, are there any other constants concerning this? As a concrete plan, it would be helpful if you could add say 2-3 sentences in the conclusion of your paper regarding the potential constants you can think of in a non-asymptotic analysis.

Reviewer #2 (Remarks to the Author):

The authors have suitably handled my comments. I would like to recommend acceptance.

We are happy to hear this! Thank you.

Reviewer #3 (Remarks to the Author):

I would like to thank the authors for the detailed replies and careful revision of the manuscript. This version is better than the initial version. However, I still have the following questions/suggestions, with "…" quoting parts the authors' reply in the rebuttal letter.

We are glad that the reviewer considers the manuscript improved.

--- "We do not think Appendix F is incremental, and we believe this required a substantial innovation of our own. Most previous work used exponentially more circuit repetitions (in N) to accurately characterize fermionic k-RDMs. Even the very recent Ref. [61], which achieves a scaling that is logarithmic in N, requires exponentially more classical post processing (in N) to estimate an element of the k-RDM than our proposed approach. The classical shadows protocol that we introduce uses similar concepts as the first work on classical shadows [57], which has resulted into many related follow-up publications in venues like Nature. Contrary to the original classical shadow approach, taking advantage of the antisymmetry of the wavefunction in first quantization demands entirely different analysis techniques and require a significant amount of work and ideas as shown in Appendix F."

My reply: I see, thanks for the argument – it is a good point that Ref. [61] requires exponentially more classical post processing time. Could you articulate the difference, and the novelty of your work, at the beginning or end of Appendix F?

We thank the referee for this suggestion. We have added two paragraphs that explain the new contributions of our classical shadows protocol, and how they succeed where past proposals have fallen short in our context, at the end of the first subsection of Appendix F.

--- "These comments also leave out many of the valuable contributions of our work. For example, Appendix A and associated discussion in the manuscript which establishes formal bounds on the cost of classical mean-field methods. No such analysis had been performed previously and allows us to compare quantum algorithms to classical mean-field algorithms on an even footing."

My reply: Regarding Appendix A, could you write a formal theorem statement with a formal proof for the scaling bound in (A14) and (A15)? Currently, the argument is not as formal as I expected. For instance, between (A10) and (A11), it simply says that "with neighboring grid points separated by δ, the smallest the average separation can be is O(η^{1/3}δ)". Why is it this specific value? I agree that norms and scaling bounds are interesting, but for the quality of Nature Communications, it would be better to present a rigorous proof here.

Our paper is generally not presented at the level of formality that the reviewer is asking for. For instance, no other appendices used formal theorem and proof statements despite presenting asymptotic scaling analysis. As to the particular question that the reviewer is asking, if we have η electrons packed into a volume of size δ^3 for each electron, the closest average separation between two electrons is O(η^{1/3}δ). This is explained in Appendix A already.

Furthermore, pg. 39 of arXiv:2211.09133 (Ref. 45) presents "formal theorem statements" that give rise to the same scaling we derive. We now mention in Appendix A that Ref. 45 also presents a formal analysis related to ours. We hope that this is a strong pointer to formal proofs that readers can easily refer to.

--- "The proposed quantum algorithms work in all regimes and there is always a quantum speedup, as shown in Figure 1. We maintain that it is quite surprising and interesting that there is ever a quantum speedup (even if sub-quadratic) when simulating systems that do not require entanglement for their description (e.g., mean-field systems). We believe this is already a significant finding."

My reply: I'm not sure if I'm following this – I think in Appendix B, you need to know the parameters in the first quantization when you apply Trotter based methods? It would be helpful if you could articulate clearly what description you require for your quantum algorithms.

The problem of sampling from the output of the quantum dynamics that we focus on in Appendix B is completely specified by the following parameters, which we regard as problem inputs and thus always known:
(1) the locations and charges of the nuclei
(2) the duration of time-evolution
(3) the number of electrons
(4) the number of basis functions
(5) the target precision to within which one realizes the correct unitary
(6) the initial state
We have added a sentence clarifying this at the start of Appendix B. This at least defines the problem of sampling from the output of the dynamics. There are further specifications of the problem that one can make regarding the observables one tries to measure. For example, in Table I, we report the scaling for several different choices of observables. Some of our quantum algorithms use a plane wave basis, and some use a grid basis (the latter is used in Appendix B). Still, the convergence of the basis error is asymptotically equivalent. So one can report an asymptotic scaling for an algorithm that solves this problem without specifying which of those representations will be employed by the algorithm.

Figure 2 analyzes the scaling with respect to the two problem size parameters: (4) and (5) above. The scaling of classical and quantum algorithms for sampling the output of the dynamics is the same in terms of (1) and is the same up to arbitrarily small polynomial or logarithmic factors in (2) and (5). Classical and quantum algorithms have different state preparation costs discussed in that

section of the paper. Still, in both cases, this state preparation cost (for mean-field states) is likely subdominant to the rest of the time evolution. That is how we can make this plot. We also now clarify in the Figure 1 caption that the assumption about state prep is the same as in Table I. Hopefully, this resolves the reviewer's concern.

--- "We also want to emphasize that a comparison to zero temperature mean-field simulations is, in this context, the cheapest simulation for a classical computer. As we emphasize in our discussion section, if the simulation is at finite temperature, or if it is a model requiring anything more than mean-field treatment, then the speedup is appreciably larger (more than quadratic in all regimes). So these findings are expected to be impactful in practice."

My reply: I feel that there is a gap here – which practical models enjoy the parameter ranges with more than quadratic speedup? Previously I asked "It would be much more helpful if the connection between the parameter range and practical models is articulated clearly", and it would be helpful that the connection to practical models is better explained.

We emphasize that mean-field simulations at finite temperatures generically give more than a quadratic speedup. We also give several examples of simulations in the warm dense and hot dense matter regimes. We added a paragraph giving further examples in our most recent revision.

At zero temperature, the level of accuracy one is targeting is an important consideration to determine the speedup. This depends much on the sorts of quantities one seeks to extract and on the particular systems and purposes. Generally, the largest speedups are found for larger basis sizes, and this occurs when higher accuracy is the goal (the relationship between N and \eta depends on precision).

An example where especially high accuracy is required is spectroscopic applications such as those arising in astrophysics studies, where the goal is often "spectroscopy accuracy" (4e-6 Hartree) instead of "chemical accuracy" (1.6e-3 Hartree). We have added mention of this to the discussion.

As our discussion now outlines both a general class of problems as well as some specific examples that are likely to have more than a quadratic speedup due to both temperature and accuracy, we feel we have addressed this comment to the extent satisfactory for initial work on the topic.

--- "There are significant length restrictions in Nature Communications and this is a relatively minor technical detail rather than something claimed as a main result. We are running up against those limits and have needed to cut content from our main body to address the reviewer's suggestion to move Figure 1 into the main body. Thus, the best solution that we came up with is to refer people to our more detailed discussion in the Appendix. If the referee has a suggestion to address this point, we would be very happy to accommodate the suggestion. Furthermore, if this is really a sticking point, it would be possible for us to remove Appendix D as this is not really our main result."

My reply: I understand that Nature Communications have a tight page limit, but this is not a reason to have a storyline not compelling enough and on the other hand have technical many appendices. The page-limit mechanism, to me, picks neat results that can be digested in a few pages. The technical contributions in the appendices of this manuscript are definitely interesting, but there's simply no space for all of them.

We agree with the reviewer about the importance of a compelling storyline and have tried to provide that to the best of our ability. In the case of this paper, it is necessary to have a number of fairly technical appendices that provide rigorous support for the narrative in the main body. One does not need to understand all of these technical details to have an appreciation of that main narrative, and that is why those details are relegated to appendices for more expert readers. The original comment that solicited the response that Reviewer 3 quoted here was about making transparent what the assumption is required for Appendix D. This is rather straightforward to explain in the main text. So we have now done that in a single sentence.

Regarding suggestions, if possible, it might be reasonable to shorten the section "Classical mean-field dynamics" a bit. It currently takes about two pages talking about bounds from different algorithms. This is probably up to the authors' discretion, but I'm not sure if all of them shall be expanded here and maybe some parts can be a bit briefer. After all, the main contribution is on the quantum computing side.

In light of the comments, the editor has graciously allowed us to slightly relax the word limit. We have thus added a number of clarifying comments on points that the reviewer has asked about during both this round of review and the former and can do so without removing too much content. Especially since the section on classical mean-field dynamics contains a number of new and important contributions, we keep the bulk of it.

Appendix D is actually okay to me, after all it's not long. I think the main point is to better present the storyline in the main body and make it cleaner.

Ok, we will keep Appendix D in that case. We have made small adjustments to try to present the main storyline better, including making it transparent in the main body that Appendix D applies only if one can soften the Coulomb potential while retaining target precision in the simulation.

--- "Our paper contains proofs on the scaling of these classical methods. Numerical simulations would perhaps help to clarify the constant factors in the classical scaling. But without knowing the constant factors in the scaling of quantum methods, it is unclear what would be gained in terms of a comparison. It would require very significant further analysis (an entirely new project) to determine the constants in the scaling of the quantum algorithms. For example, the constant factors in the number of Trotter steps required in this representation is unknown and difficult to estimate numerically. Other classical methods that we did not analyze in this paper are only more costly with a worse scaling than the classical methods we compare to in our paper (mean-field methods). Thus, our quantum algorithms would have large polynomial speedups over such

methods in all regimes. This, therefore, does not affect the main conclusion of our paper and only strengthens the conclusion of the paper. We do note this aspect in the conclusion of our paper.”

My reply: I very much agree that it requires significant further effort to determine the constants in the scaling of the quantum algorithms. Nevertheless, maybe the authors can at least make a bit more efforts and let the readers know where the potential overheads can come from? This reply mentions the constant factors in the number of Trotter steps, are there any other constants concerning this? As a concrete plan, it would be helpful if you could add say 2-3 sentences in the conclusion of your paper regarding the potential constants you can think of in a non-asymptotic analysis.

The cost of time-evolution is just the number of Trotter steps required to perform a simulation to a given target error and time, multiplied by the cost of each Trotter step. To first order (i.e., to correctly estimate the cost to within perhaps 10% of the total Toffoli gates), the cost of the Trotter step is dominated by the computation of the Coulomb operator (computing the sum of eta^2 terms of form 1 / |r_i - r_j|). This part is rather straightforward. For a concrete (albeit outdated) reference that addresses the constant factors in the Toffoli complexity of this step, one can see Section 4 of arXiv:1204.0567. We now point this out and cite that work in Appendix B.

The bigger source of uncertainty regarding these constant factors is in the requisite Trotter number. The requisite Trotter number, in this case, is known to scale as stated in our manuscript, but the constant factors in Trotter errors are notoriously difficult to pin down. Examples of papers performing this sort of analysis are arXiv:1406.4920, arXiv:1410.8159, arXiv:1711.10980, and arXiv:1902.10673. In all cases, the finding is that the constant factors scaling the Trotter errors tend to be quite low (sometimes chemical accuracy is possible with just hundreds or thousands of Trotter steps for classically intractable systems). However, a significant amount of work is needed to determine those constant factors within a couple of orders of magnitude. Given this, we do not feel comfortable speculating on these values.

REVIEWERS' COMMENTS

Reviewer #3 (Remarks to the Author):

I would like to thank the authors for the detailed responses and revisions of the manuscript.

I'm happy to open my green light for this paper at Nature Communications now.

From my perspective, this is probably still not like a best paper for Nature Communications, but it is a solid result, and the issues I noticed had been addressed throughout the two rounds of revisions.

As a last point, I would still insist that a bit more discussion about the potential constants in a non-asymptotic analysis can be helpful. To me, research papers should state not only advantages but also limitations. I guess the authors shouldn't feel ashamed that the constant factors scaling the Trotter errors tend to be quite low but a significant amount of work is needed to determine those constant factors within a couple of orders of magnitude. Leaving this as future work and make a bit connection to arXiv:1406.4920, arXiv:1410.8159, arXiv:1711.10980, and arXiv:1902.10673 should be beneficial for the readers.

Reviewer #3 (Remarks to the Author):

I would like to thank the authors for the detailed responses and revisions of the manuscript. I'm happy to open my green light for this paper at Nature Communications now. From my perspective, this is probably still not like a best paper for Nature Communications, but it is a solid result, and the issues I noticed had been addressed throughout the two rounds of revisions.

We are very happy that the reviewer agrees our paper is now suitable for publication in Nature Communications. We agree this correspondence has improved the manuscript.

As a last point, I would still insist that a bit more discussion about the potential constants in a non-asymptotic analysis can be helpful. To me, research papers should state not only advantages but also limitations. I guess the authors shouldn't feel ashamed that the constant factors scaling the Trotter errors tend to be quite low but a significant amount of work is needed to determine those constant factors within a couple of orders of magnitude. Leaving this as future work and make a bit connection to arXiv:1406.4920, arXiv:1410.8159, arXiv:1711.10980, and arXiv:1902.10673 should be beneficial for the readers.

We have added two sentences to Section 2B that address this. Below Equation 12 we write:

While efficient explicit circuits such as those in [46] can be used to perform Trotter steps in this representation, more work would be required to determine the constant factors associated with the number of Trotter steps required. Prior analyses of the requisite Trotter number for chemistry have generally found that constant factors are low, but focused on different representations or lower order formulas [47-51].
